# Bacterial Isolate Inhabiting Spitsbergen Soil Modifies the Physiological Response of *Phaseolus coccineus* in Control Conditions and under Exogenous Application of Methyl Jasmonate and Copper Excess

**DOI:** 10.3390/ijms20081909

**Published:** 2019-04-17

**Authors:** Agnieszka Hanaka, Artur Nowak, Andrzej Plak, Sławomir Dresler, Ewa Ozimek, Jolanta Jaroszuk-Ściseł, Magdalena Wójciak-Kosior, Ireneusz Sowa

**Affiliations:** 1 Department of Plant Physiology, Maria Curie-Skłodowska University, Akademicka St. 19, 20-033 Lublin, Poland; slawomir.dresler@poczta.umcs.lublin.pl; 2 Department of Environmental Microbiology, Maria Curie-Skłodowska University, Akademicka St. 19, 20-033 Lublin, Poland; artur.nowak@poczta.umcs.lublin.pl (A.N.); ozimek@poczta.umcs.lublin.pl (E.O.); jolanta.jaroszuk-scisel@poczta.umcs.lublin.pl (J.J.-Ś.); 3 Department of Geology and Soil Science, Maria Curie-Skłodowska University, Kraśnicka Ave. 2cd, 20-718 Lublin, Poland; aplak@poczta.umcs.lublin.pl; 4 Department of Analytical Chemistry, Medical University of Lublin, Chodźki 4a, 20-093 Lublin, Poland; magdalena.wojciak-kosior@umlub.pl (M.W.-K.); ireneuszsowa@umlub.pl (I.S.)

**Keywords:** allantoin, bacterial isolate, CAT, copper, organic acids, PAL, peroxidase, TAL, secondary metabolites, SOD

## Abstract

The aim of the study was to demonstrate the potential of the promotion and regulation of plant physiology and growth under control and copper stress conditions, and the impact of the exogenous application of methyl jasmonate on this potential. Runner bean plants were treated with methyl jasmonate (1 or 10 µM) (J; J1 or J10) and Cu (50 µM), and inoculated with a bacterial isolate (S17) originating from Spitsbergen soil, and identified as *Pseudomonas luteola* using the analytical profile index (API) test. Above- and under-ground plant parts were analyzed. The growth parameters; the concentration of the photosynthetic pigments, elements, flavonoids (FLAVO), phenolics (TPC), allantoin (ALLA), and low molecular weight organic acids (LMWOAs); the activity of antioxidant enzymes and enzymes of resistance induction pathways (e.g., superoxide dismutase (SOD), catalase (CAT), ascorbate (APX) and guaiacol (GPX) peroxidase, glucanase (GLU), and phenylalanine (PAL) and tyrosine ammonia-lyase (TAL)), and the antioxidant capacity (AC) were studied. The leaves exhibited substantially higher ALLA and LMWOA concentrations as well as PAL and TAL activities, whereas the roots mostly had higher activities for a majority of the enzymes tested (i.e., SOD, CAT, APX, GPX, and GLU). The inoculation with S17 mitigated the effect of the Cu stress. Under the Cu stress and in the presence of J10, isolate S17 caused an elevation of the shoot fresh weight, K concentration, and TAL activity in the leaves, and APX and GPX (also at J1) activities in the roots. In the absence of Cu, isolate S17 increased the root length and the shoot-to-root ratio, but without statistical significance. In these conditions, S17 contributed to a 236% and 34% enhancement of P and Mn, respectively, in the roots, and a 19% rise of N in the leaves. Under the Cu stress, S17 caused a significant increase in FLAVO and TPC in the leaves. Similarly, the levels of FLAVO, TPC, and AC were enhanced after inoculation with Cu and J1. Regardless of the presence of J, inoculation at Cu excess caused a reduction of SOD and CAT activities, and an elevation of GPX. The effects of inoculation were associated with the application of Cu and J, which modified plant response mainly in a concentration-dependent manner (e.g., PAL, TAL, and LMWOA levels). The conducted studies demonstrated the potential for isolate S17 in the promotion of plant growth.

## 1. Introduction

Elicitors are chemicals able to trigger morphological and physiological responses in the plant organism, activating or reducing a defined set of compounds [1]. Among the elicitors, methyl jasmonate (J) is an important naturally occurring plant growth regulator that modulates plant status by activation of the signal transduction pathways [2]. Jasmonate acid (JA), just as ethylene, is involved in the induction of systemic resistance (ISR), which is mediated by plant growth-promoting bacteria (PGPB) capable of stimulating plant growth [3]. Being involved in plant signaling pathways, J also participates in the generation of highly reactive oxygen species (ROS), which stimulate the plant defense system by the production of various enzymatic and non-enzymatic antioxidant mechanisms. Detoxifying enzymes—superoxide dismutase (SOD), which decomposes superoxide into hydrogen peroxide, and catalase (CAT) with ascorbate (APX) and guaiacol (GPX) peroxidases, which convert hydrogen peroxide into water—represent the first line of defense for lowering the level of ROS [4]. To study its potential in the plant organism, J can be applied exogenously [5,6,7]. 

The second line of defense consists of endogenous antioxidative chemicals of different sources, namely: carotenoids (Car), flavonoids (FLAVO), phenolics (TPC), and the substrates of antioxidative enzymes (e.g., ascorbate or guaiacol for APX or GPX, respectively) [4]. Secondary metabolites (SM) may play distinct roles. For example, TPC can serve as chelators [8], compounds with antioxidant activity (due to the presence of hydroxyl groups, which are responsible for protecting biomembranes from peroxidation injuries) [9], and donors of electrons for GPX, or they can react with FLAVO [4].

Among the defense-related enzymes, are phenylalanine ammonia-lyase (PAL) and tyrosine ammonia-lyase (TAL), which are involved in plant stress responses via phenylpropanoid metabolism, leading to the production of defensive compounds (e.g., FLAVO) [10], which are also useful in plant–pathogen interaction. PAL converts L-phenylalanine to trans-cinnamic acid, and TAL converts l-tyrosine to ammonia and *p*-coumaric acid [11]. PAL activity induced by the microbial antagonists in hosts was positively related to their biocontrol effects [12].

Pathogenesis-related (PR) proteins are responsible for defense responses and can be induced by the application of such chemicals as J [13]. Among PR proteins, β-1,3-glucanase (GLU), which is a PR-2 protein [13], hydrolyses the β-*O*-glycosidic bond of β-glucan in plant and fungal cell walls [14] and can accumulate in biotic and abiotic stress conditions [13].

Another group of metabolites that may serve as ROS scavengers protecting plants from the oxidative damage of different stresses are intermediates of the ureide metabolism known in legumes and non-legume plants [15]. Allantoin (ALLA; 5-ureidohydantoin) is generated from uric acid. ALLA, a heterocyclic N compound derived from purine, plays a pivotal role in the assimilation, metabolism, transport, and storage of N in plants, and participates in chemical interactions between plants [16]. ALLA synthesized in the nodules is transported to the underground parts, where its degradation and N re-assimilation takes place [17]. Its main advantage as a N-transport molecule is the high N:C ratio of 1:1, which expresses the minimal expense of reduced C while providing N to the plant [18]. 

In plants, low molecular weight organic acids (LMWOAs) are important root exudates formed mainly from the mitochondrial tricarboxylic acid cycle, and partially from intermediate products of the glyoxylate cycle [19]. Among them, tartrate, malate, citrate, or succinate serve as natural extracellular and intracellular chelators and sequestrators of metals in the vacuole, cell wall, or trichomes, leading to metal tolerance or detoxification [6,20,21,22]. Therefore, there are attempts to apply exogenous LMWOAs in the decontamination of soil [23]. 

As an essential micronutrient, Cu is beneficial for living organisms only in a narrow range of concentrations, and is toxic at high levels [24]. The Cu concentration in the soil depends on the type and mineral properties of the parent material, the soil formation process, and anthropogenic activity (e.g., agriculture, traffic, mining, and domestic heating).

Soil microorganisms play a key role in the recycling of soil macro- and micronutrients, making them available to plants [25]. Via direct and indirect mechanisms of metal immobilization, they are the main factors determining the distribution of metals among other microorganisms inhabiting the soil and plants [26,27]. The mechanisms include biosorption and bioaccumulation by biomass, the synthesis of exopolymers, and the complexation of metals through microbiological chelators, incorporating the release of organic acids and specific organic chelators with a low molecular weight, characterized by a very high affinity for Fe (III) (>10^30^·M^−1^) and many divalent cations. Cu is a divalent transition metal, for which siderophores exhibit the highest affinity [28]. The microbial production of siderophores, and the ability to synthesize phytohormones such as indoleacetic acid (IAA) and to solubilise phosphorus, are a mechanism of plant growth promotion (PGP) by microorganisms. The mechanism not only improves plant growth, but also decreases the negative impact of toxic heavy metal concentrations and increases plant resistance to metal stress [29,30]. Thus, PGP microorganisms are of fundamental importance in determining plant–soil–metal associations [31].

The growth of plants used for the phytostabilization of moderately copper-contaminated soils (e.g., *Vicia faba*) has been elevated by 30% after soil inoculation with appropriate PGPB [32]. It can also be enhanced by more than 300% in the presence of Cu-tolerant bacteria [33].

In the family Fabaceae (Leguminosae), the key cultivated species among *Phaseolus* is runner bean (*Phaseolus coccineus* L.), which is commonly grown in Europe and America for its seeds, which are especially rich in proteins, as well as carbohydrates, mineral salts, and vitamins from group B [34]. *P. coccineus* acts as a model dicotyledonous plant in physiological research [5,6].

To the best of our knowledge, there are limited data about the influence of bacterial isolates that are adapted to cold stress, and are capable of growing in a wide range of temperatures (psychrotrophs) on plant physiology and biochemistry. A source of such strains is Spitsbergen soil. In our previous research, we screened a broad range of isolates from Spitsbergen soils in search of those that are promising in stimulating plant growth [35] and we chose one of them. As bacterial isolates from Spitsbergen seem to have potential for PGP and evoking resistance in stress conditions, it is agriculturally and environmentally valuable to verify their ability to influence the plant status in the presence of exogenously applied J and Cu in excess. Some scientists have evaluated Cu-tolerant rhizobacteria for plant growth promotion [36]. We hypothesize that these bacterial isolates may have the ability to modify plant response in control and stress conditions, which changes plant metabolism or favors the stimulation of plant growth. 

The aim of the study was to provide physiological and biochemical insight into the potential of the selected bacterial isolate (*Pseudomonas luteola*—based on the analytical profile index (API) test and named S17) originating from Spitsbergen soil in the promotion of *P. coccineus* growth and its ability to modify plant response under Cu excess and in the presence of J. The specific objectives of the study were (I) to examine the plant growth parameters and concentrations of the pigments and elements; (II) to test the FLAVO and TPC concentration, antioxidant capacity, and the activity of the antioxidant- and defense-related enzymes; (III) to study the allantoin and LMWOA concentrations; and finally, (IV) to elucidate the potential of the isolate in modifying *P. coccineus* response. 

## 2. Results

### 2.1. Phenotypic Characteristics of the S17 Isolate 

**Figure 1:**
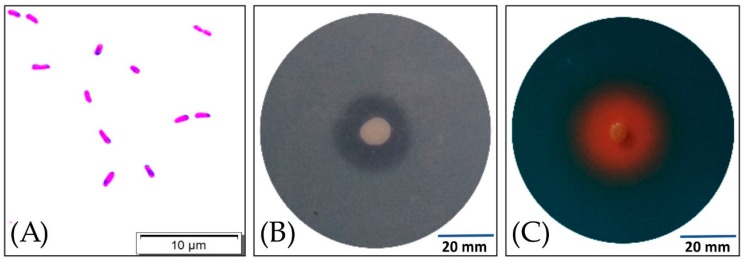
Image of Gram-negative rod-shaped cells of the S17 isolate under light microscopy (**A**). Clear halo-zone of phosphate solubilization around the S17 isolate colony on a Pikovskaya (PVK) modified medium after 48 h of inoculation at 20 °C (**B**). Orange halo-zone around the colony of S17 isolate after 24 h of inoculation at 20 °C on the chrome azurol S blue medium (**C**).

In our previous studies [35], the S17 isolate was biochemically identified as Gram-negative (Figure 1A) *Pseudomonas luteola*, and characterized by growth on oligotrophic and coptotrophic media, the activity of ACC (1-aminocyclopropane-1-carboxylate) deaminase, the concentration of IAA, and the Minimal Inhibitory Concentration (MIC) for Cu. We also obtained positive results in the following tests: the activity of protease, β-glucosidase, and β-galactosidase; the reduction of nitrate to nitrite ions; the fermentation of d-glucose, the hydrolysis of d-glucose, l-arabinose, d-mannose, d-mannitol, *N*-acetylglucosamine, d-maltose, gluconate, malate, and citrate; phosphate solubilization on Pikovskaya (PVK) medium (Figure 1B); and the production of siderophores on blue agar with chrome azurol S medium (Figure 1C). The S17 isolate stimulated the germination of *Phaseolus coccineus* seeds to a slight degree (121.2 ± 13.9% of the control).

Moreover, the capability of the biofilm formation was determined. The effect of Cu on the growth and activity of the S17 isolate in cultures with Cu (50 µM) and without metal addition was studied. Also, the ability of the S17 isolate to synthesize IAA, phenolic and Fe(III) complexing compounds, hydroxamate, and catechole siderophores was determined using the following three media: (1) minimal medium (MM9) with 0.4% glucose, (2) MM9 with 0.4% sucrose, and (3) Luria–Bertani broth (LB). The results are presented in Table 1.

The S17 isolate grew even ca. 100 times more intensely on the rich LB medium than on MM9. The addition of Cu caused a 2–5-fold and 10–20-fold inhibition of growth on the MM9 medium with glucose and sucrose, respectively. However, on the LB medium, the addition of 50 μM Cu did not inhibit growth. The isolate alkalized the rich substrate and acidified the poor one. The addition of tryptophan (Trp) to the MM9 medium with glucose and sucrose stimulated the growth of 24 h cultures by approximately 40% and 130%, respectively, and did not affect the growth of the isolate on the rich LB medium. Regardless of the C source, the isolate was capable of producing phytohormone IAA and various metal complexing compounds. Although IAA reached the highest concentration in the 48 h LB culture, the strongest effect of the Trp precursor was observed on MM9 with glucose, and the phytohormone synthesis was independent of Cu. The isolate demonstrated the ability to synthesize Fe(III) complexing compounds on both of the tested media, with a particularly high total number of siderophores detected in the CAS test on the LB media. Trp stimulated and Cu inhibited the ability to synthesize the hydroxamate siderophores, but the synthesis of the catechole siderophores with the highest affinity for metals was independent of Trp and Cu, as well as the synthesis of phenolic compounds.

### 2.2. Plant Growth Parameters

**Figure 2:**
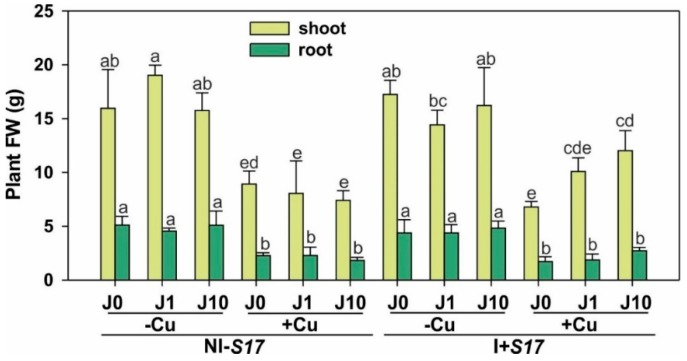
Biomass of *Phaseolus coccineus* shoots and roots. Data are means ± SD (*n* = 30). Values for shoots and roots followed by the same letter are not significantly different (*p* < 0.05; Tukey’s test). Abbreviations: J0 = without methyl jasmonate (J); J1 = with 1 µM of J; J10 = with 10 µM of J; −Cu = without Cu; +Cu = with 50 µM Cu; NI − S17 = no inoculation with the S17 isolate; I + S17 = inoculation with the S17 isolate; FW = fresh weight.

Irrespective of the inoculation with isolate S17 (I + S17) and the presence of J, the plant biomass was significantly reduced after Cu application, but I + S17 improved the shoot biomass by 62.5% under Cu and J10 supplementation, compared to the treatment that was non-inoculated with isolate S17 (NI − S17) (Figure 2 and Table S1). Furthermore, the Cu supplementation reduced the plant biomass. The shoot-to-root ratio did not change, except for the increase by 52.6% in the I + S17 variant with Cu addition and J1 application, compared to NI − S17 (Figure S1). Compared to NI − S17, the isolate caused a reduction of the root length in the J environment, regardless of its concentration. Inoculation with subsequent Cu and J10 application increased the root length by 40.4% compared to plants NI − S17. Moreover, irrespective of the inoculation, the Cu addition decreased the root length.

### 2.3. Content of Photosynthetic Pigments

**Figure 3:**
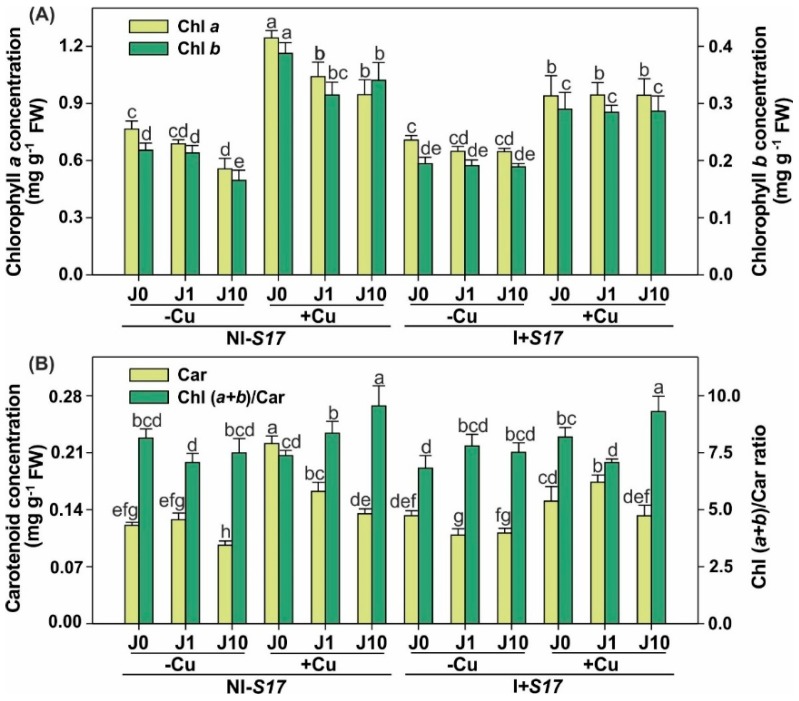
Concentration of photosynthetic pigments in *Phaseolus coccineus* leaves: chlorophylls *a* and *b* (**A**); carotenoids (Car) and the ratio of the sum of chlorophylls to carotenoids (Chl (*a+b*)/Car) (**B**). Data are means ± SD (*n* = 3). Values followed by the same letter are not significantly different (*p* < 0.05; Tukey’s test). Abbreviations: J0 = without methyl jasmonate (J); J1 = with 1 µM of J; J10 = with 10 µM of J; −Cu = without Cu; +Cu = with 50 µM Cu; NI − S17 = no inoculation with the S17 isolate; I + S17 = inoculation with the S17 isolate; FW = fresh weight.

Inoculation (without Cu supplementation) had no effect on the concentration of both chlorophylls (Chls), regardless of the presence of J, compared to NI − S17. In turn, I + S17 with the Cu application declined the concentration of Chls in plants that were not exposed to J, and lowered the chlorophyll *b* (Chl *b*) level in plants supplemented with J10, compared to NI − S17 (Figure 3A and Table S1). Regardless of the inoculation, the Cu addition elevated the concentration of Chls. Compared to NI − S17, in the I + S17 variant without Cu, the Car concentration increased after the addition of J10, and decreased in the treatment with Cu without addition of J (Figure 3B). In NI − S17, the Cu application increased the Car concentration, and in the I + S17 variant, Cu elevated the Car level at the J1 application. In spite of the inoculation, the highest Chl-to-Car ratio was noted in the treatment with Cu and J10.

Irrespective of the inoculation, the sum of Chls increased after the Cu supplementation in comparison with the treatments without Cu (Figure S2, Table S1). Inoculation with the Cu addition, but without J, reduced the sum of Chls, compared to NI − S17. In the environment of J10 and Cu, the chlorophyll *a* (Chl *a)*/*b* ratio was elevated in the I + S17 treatment.

### 2.4. Content of Elements

**Figure 4:**
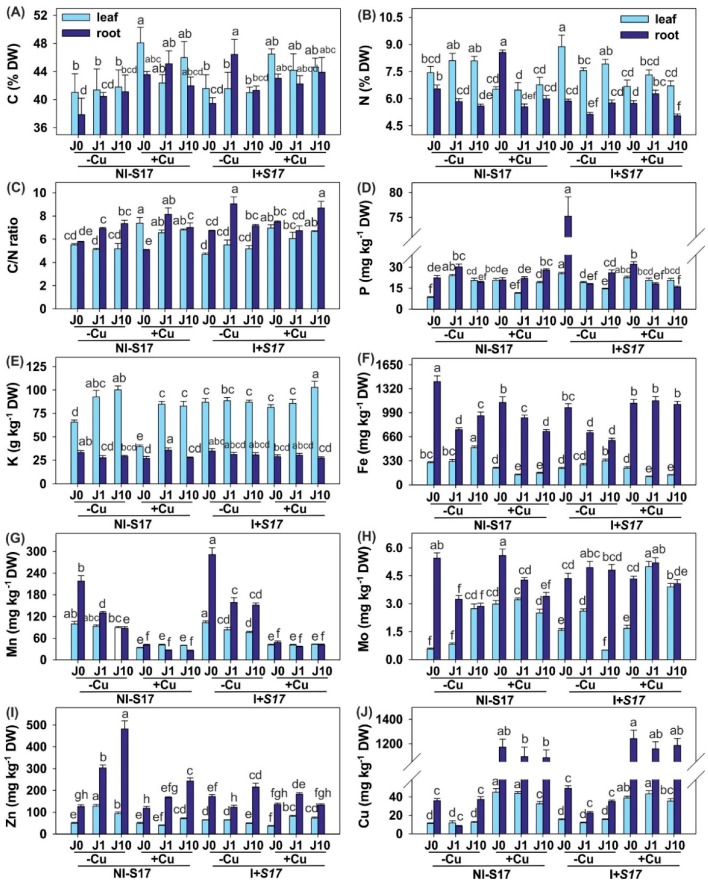
Concentration of selected elements in *Phaseolus coccineus* leaves and roots: C (**A**); N (**B**); C/N (**C**); P (**D**); K (**E**); Fe (**F**); Mn (**G**); Mo (**H**); Zn (**I**); and Cu (**J**). Data are means ± SD (*n* = 3). Values for leaves and roots followed by the same letter are not significantly different (*p* < 0.05; Tukey’s test). Abbreviations: J0 = without methyl jasmonate (J); J1 = with 1 µM of J; J10 = with 10 µM of J; −Cu = without Cu; +Cu = with 50 µM Cu; NI − S17 = no inoculation with the S17 isolate; I + S17 = inoculation with the S17 isolate; DW = dry weight.

Generally, the C, N, and K contents were higher in the shoots than in the roots (Figure 4A,B,E and Table S1). Overall, the C/N ratio and the concentrations of P, Fe, Mn, Mo, Zn, and Cu were elevated in the roots (Figure 4C,D,F–J). 

The highest concentrations of N (19% increase), P, and Mn were detected in the leaves, and the highest levels of P (236% enhancement) and Mn (34% elevation) were determined in the roots in variant I + S17 (without J and Cu). Regardless of the inoculation, the Cu addition resulted in statistically the same level of Cu accumulation in the plant tissues. The application of J led to a reduction of N, Fe, Mn, and Mo, and an elevation of Zn in the roots. 

In the leaves of the NI − S17 (non-J-), but Cu-treated plants, the concentrations of C, P, and Mo were higher and the levels of K and Fe were lower than in the plants that were not supplemented with Cu. In turn, the levels of C and N in the roots were elevated, whereas the concentrations of K and Fe were reduced, compared with the Cu-untreated plants. The leaves of the NI − S17 J1- and Cu-supplemented plants showed a rise in the Mo concentration and a decline in the N, P, Fe, and Zn accumulation, compared with the Cu-untreated plants. The roots of these plants exhibited elevated C, K, Fe, and Mo levels, simultaneously with decreased P and Zn concentrations, compared to treatments without Cu addition. In the leaves of NI − S17 J10- and Cu-treated plants, the concentrations of N, K, Fe, and Zn declined, compared with the non-Cu-supplemented plants. Moreover, regardless of the inoculation, after the Cu application, the concentration of Mn was lower, whereas the level of Cu was higher in both the leaves and roots.

The inoculation of the plants with the S17 isolate with Cu addition resulted in a reduction in Zn accumulation in the leaves and N in the roots, compared to the non-Cu-supplemented plants. The concentrations of P, Mo, and Zn increased in the leaves, whereas the concentrations of N, Fe, and Mo rose in the roots of these plants with the J1 addition. The elevation of the K and Mo concentrations was determined in the leaves, whereas an increase in Fe and a decrease in N and P concentrations were noted in the roots of the plants with the J10 addition.

The concentration of N, P, K, Mo, and Zn was higher, while the level of Fe was lower in the inoculated (non-J- and non-Cu-treated) leaves compared with NI − S17. In turn, in the roots of these plants, the P, Mn, and Zn concentrations were elevated, whereas the content of N, Fe, and Mo declined. 

The Mo level rose, while the concentration of P and Zn was reduced in the leaves of J1 supplemented (but non-Cu-treated) plants, whereas the concentrations of C, Mn, and Mo in the roots increased simultaneously with a decrease in the N, P, and Zn levels, compared with NI − S17. In the I + S17 treatment with the application of J10 (but without the presence of Cu), the leaves exhibited a reduced P, K, Fe, Mn, Mo, and Zn accumulation, whereas the roots were characterized by a rise in the P, Mn, and Mo levels, concurrently with a decline in Fe and Zn levels.

The K level increased, whereas the content of Mo and Zn declined in the leaves of the I + S17 and Cu-supplemented plants; the accumulation of P in the roots was higher, but that of N and Mo was lower compared to NI − S17. In this treatment, after J1 addition, the P, Mo, and Zn levels were elevated in the leaves, and N, Fe, and Mo were accumulated in the roots. Under the J10 supplementation, the K and Mo levels in the leaves increased and the roots exhibited an increase in the Fe concentration and a decline in N, P, and Zn. 

### 2.5. Content of Flavonoids and Phenolics and the Level of Antioxidant Capacity

Generally, the Cu excess resulted in an elevation of FLAVO and TPC concentrations and antioxidant capacity, in both 2-azino-bis-3-ethylbenzthiazoline-6-sulphonic acid (ABTS) and 2,2-diphenyl-1-picrylhydrazyl (DPPH) tests (Table 2 and Table S2). 

Regardless of the plant part, NI − S17 in Cu excess elevated FLAVO, TPC, and antioxidant capacity, compared to the variant without Cu. The J1 addition to this treatment equaled, increasing the TPC and ABTS values in both the leaves and the roots, and the FLAVO and DPPH values were higher only in the roots. The J10 supplementation of this treatment generated increases in all of the analyzed values, except for ABTS in the leaves.

In spite of the J application, I + S17 in the Cu environment elevated all of the measured parameters in all of the treatments, except for DPPH in the leaves (I + S17 + J10 + Cu), compared with the treatments without Cu.

The inoculation with the J10 addition produced lower TPC, ABTS, and DPPH values in the leaves, and the TPC and ABTS levels were higher only in the roots, compared with NI − S17.

Higher results were noted for FLAVO and TPC in the leaves, and lower values were determined for TPC and antioxidant capacity in the roots, in the I + S17 treatment with the Cu application compared with NI − S17. The addition of J1 to the above-mentioned treatment caused an elevation of the values of all of the parameters, except for TPC in the roots and ABTS in the leaves. However, after the J10 addition, the plants showed lower levels of FLAVO in the roots, TPC in the leaves, and DPPH in the leaves and roots, compared with NI − S17. 

### 2.6. Activity of Antioxidant Enzymes—Plant Resistance Markers

**Figure 5:**
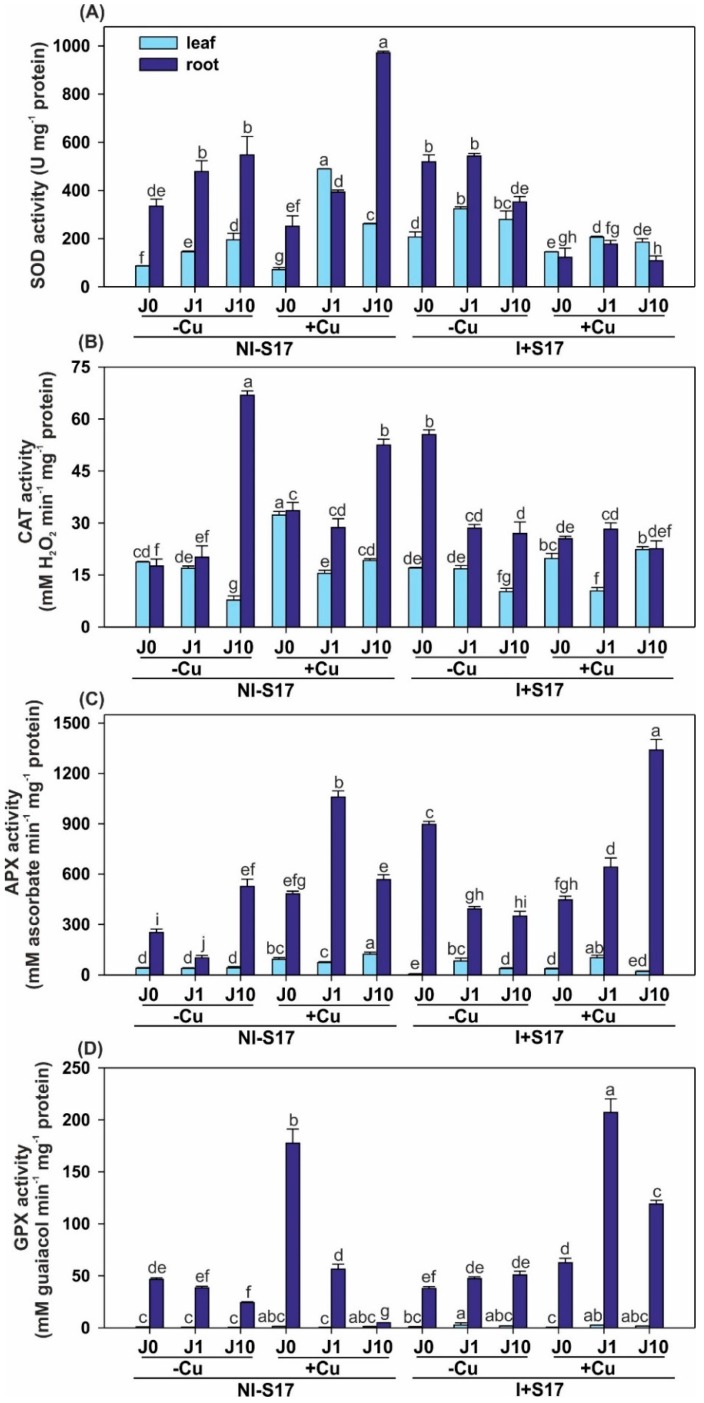
Activity of antioxidative enzymes in *Phaseolus coccineus* leaves and roots, as follows: superoxide dismutase (SOD) (**A**); catalase (CAT) (**B**); ascorbate peroxidase (APX) (**C**); guaiacol peroxidase (GPX) (**D**). Data are means ± SD (*n* = 3). Values for the leaves and roots followed by the same letter are not significantly different (*p* < 0.05; Tukey’s test). Abbreviations: J0 = without methyl jasmonate (J); J1 = with 1 µM of J; J10 = with 10 µM of J; −Cu = without Cu; +Cu = with 50 µM Cu; NI − S17 = no inoculation with the S17 isolate; I + S17 = inoculation with the S17 isolate.

The enzymatic activity was higher in the roots than in the leaves of *P. cocccineus* plants (Figure 5 and Table S2). In the NI − S17 (and non-Cu-treated plants), the J application elevated SOD activity in both the leaves and roots; additionally, the higher J concentration enhanced the CAT and APX levels, but reduced GPX in the roots. Similar effects were detected in NI − S17 with the Cu addition (except for APX).

In the NI − S17, Cu-supplemented plants, the activity of CAT and APX was elevated in the leaves, and higher CAT, APX, and GPX activities were detected in the roots, compared with the treatments without Cu. In this treatment, J1 addition increased the activity of SOD and APX in the leaves. In turn, the activity of CAT and of both peroxidases was stimulated, and that of SOD was reduced in the roots. The supplementation of J10 enhanced the activities of SOD, CAT, and APX in the leaves, whereas the SOD level rose and the CAT and GPX activity declined in the roots. 

Regardless of the plant part and the presence of J, the activity of SOD decreased in the I + S17 and Cu supplementation treatment, compared with the variant without Cu. The inoculation with Cu excess raised the CAT and APX activities in the leaves, but lowered these parameters in the roots, and the GPX level in the roots increased. The supplementation with J1 reduced the SOD and CAT activity in the leaves and increased the activity of both of the peroxidases.

In the bacterial isolate-treated plants, the SOD activity rose, and that of APX declined in the leaves, whereas the SOD, CAT, and APX levels increased in the roots, compared with NI − S17. The addition of J1 increased the activity of SOD and peroxidases in the leaves, and CAT and APX in the roots. The J10 supplementation raised the SOD level in the leaves, while in the roots, the SOD, CAT, and APX activities were lower and GPX was enhanced.

The inoculation with Cu addition increased the SOD activity and reduced the CAT and APX levels in the leaves, compared with NI − S17. The activities of SOD, CAT, and GPX declined in the roots. In the leaves, in the same treatment with the addition of J1, the activities of SOD and CAT were reduced, while the level of peroxidases rose. In the roots, the above-mentioned variant with J1 produced a reduction in the levels of SOD and APX, whereas GPX increased (3.7-fold). In the leaves of the plants from the same treatment, at the presence of J10, the SOD and APX levels were reduced, but CAT increased. In the roots, J10 caused a reduction in the activities of SOD and CAT, and an enhancement in the activity of the peroxidases (APX: 2.4-fold and GPX: 24.5-fold). 

### 2.7. Activity of Defence-Related Enzymes—Plant Resistance Markers

**Figure 6:**
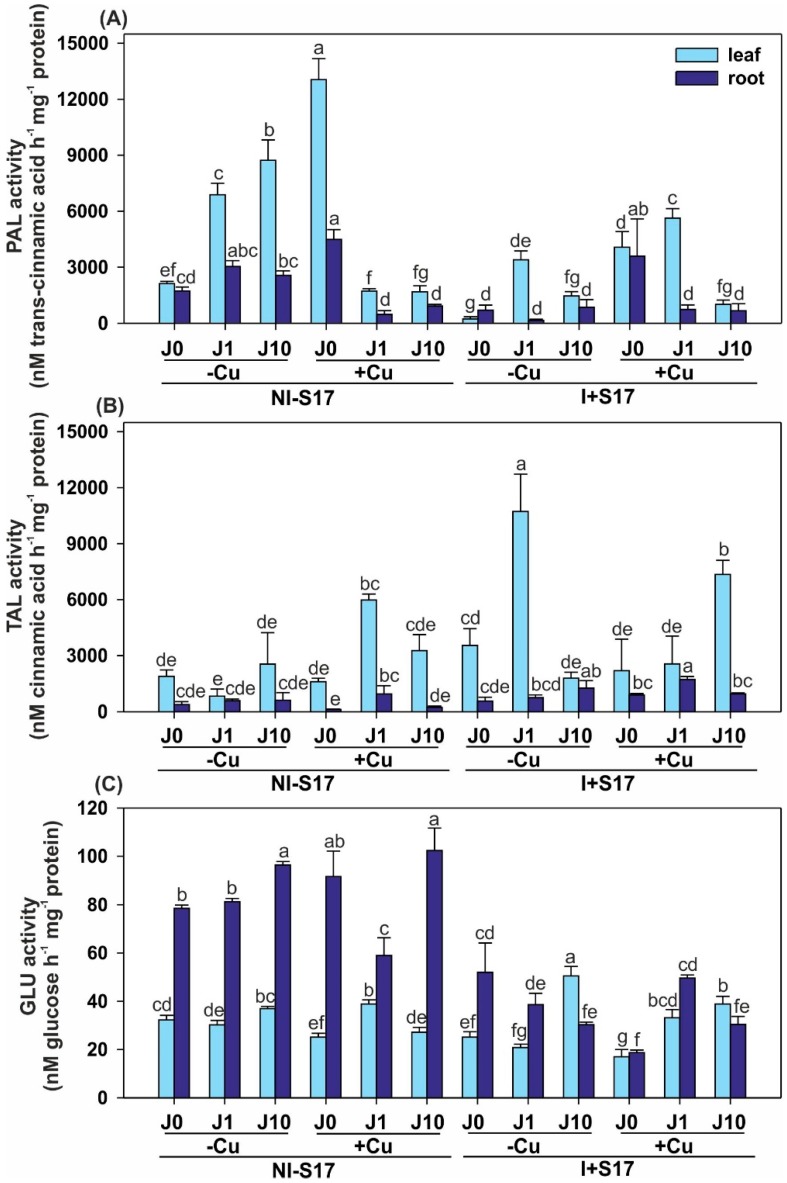
Activity of defence-related enzymes—plant resistance markers in *Phaseolus coccineus* leaves and roots, namely: phenylalanine ammonia-lyase (PAL) (**A**); tyrosine ammonia-lyase (TAL) (**B**); β-1,3-glucanase (**C**). Data are means ± SD (*n* = 3). Values for the leaves and roots followed by the same letter are not significantly different (*p* < 0.05; Tukey’s test). Abbreviations: J0 = without methyl jasmonate (J); J1 = with 1 µM of J; J10 = with 10 µM of J; −Cu = without Cu; +Cu = with 50 µM Cu; NI − S17 = no inoculation with the S17 isolate; I + S17 = inoculation with the S17 isolate.

Overall, PAL and TAL showed higher activity in the leaves, while GLU levels were higher in the *P. coccineus* roots (Figure 6 and Table S1).

In NI − S17, the Cu excess caused a rise in PAL and a decline in GLU activities in the leaves and roots, compared with the non-Cu-supplemented variant. The addition of J1 increased the TAL and GLU levels and decreased PAL in the leaves. In the roots, the levels of PAL and GLU were reduced. The J10 treatment lowered the activities of GLU and PAL in the leaves, and PAL in the roots.

The inoculation with Cu elevated PAL activity in the leaves and roots, compared with the treatments without Cu. The supplementation with J1 increased the levels of PAL and GLU, and reduced TAL in the leaves, while TAL activity grew in roots. The additional J10 application increased the TAL level and decreased GLU.

The bacterial isolate lowered PAL and GLU activities in the leaves and GLU in the roots, compared with NI − S17. The addition of J1 decreased the PAL and GLU levels in both the leaves and roots, and elevated TAL activity in the leaves. The treatment with J10 reduced the PAL activity and elevated GLU in the leaves. In turn, PAL and GLU decreased and TAL enhanced in the roots.

After the inoculation with Cu excess, the activities of PAL and GLU were reduced in the leaves, but elevated TAL and lowered GLU levels were determined in the roots, compared with NI − S17. The application of J1 increased the PAL activity (3.3-fold) and reduced TAL in the leaves, whereas the roots exhibited increased levels of this enzyme. The supplementation with J10 increased the TAL (2.2-fold) and GLU activities in the leaves. In turn, TAL was higher and GLU was lower in the roots. 

### 2.8. Content of Allantoin

**Figure 7:**
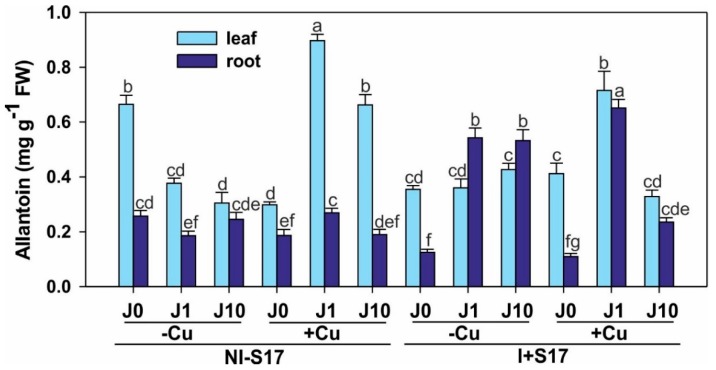
Concentration of allantoin in *Phaseolus coccineus* leaves and roots. Data are means ± SD (*n* = 3). Values for the leaves and roots followed by the same letter are not significantly different (*p* < 0.05; Tukey’s test). Abbreviations: J0 = without methyl jasmonate (J); J1 = with 1 µM of J; J10 = with 10 µM of J; −Cu = without Cu; +Cu = with 50 µM Cu; NI − S17 = no inoculation with the S17 isolate; I + S17 = inoculation with the S17 isolate; FW = fresh weight.

ALLA was elevated in the leaves, compared with the roots, except for the treatments with both I + S17 and J (without Cu application) (Figure 7 and Table S3). 

In the NI − S17 variant, the Cu addition lowered the ALLA level in the leaves and roots at J0, and a decline in ALLA content was noted at J1 and J10 (except J10 in the roots), compared with the non-Cu-supplemented treatment. The inoculation with Cu elevated the ALLA level at J1 in the leaves and roots, and decreased it at J10 in the roots.

After the inoculation, the ALLA concentration declined at J0, but increased at J1 and J10 (except no difference at J1 in the leaves), compared with NI − S17. The addition of Cu resulted in a higher ALLA concentration in the leaves at J0, lower at J1 (of 20.3%) and J10, and elevated in the roots at J1 (2.4-fold). 

### 2.9. Content of LMWOAs

**Figure 8:**
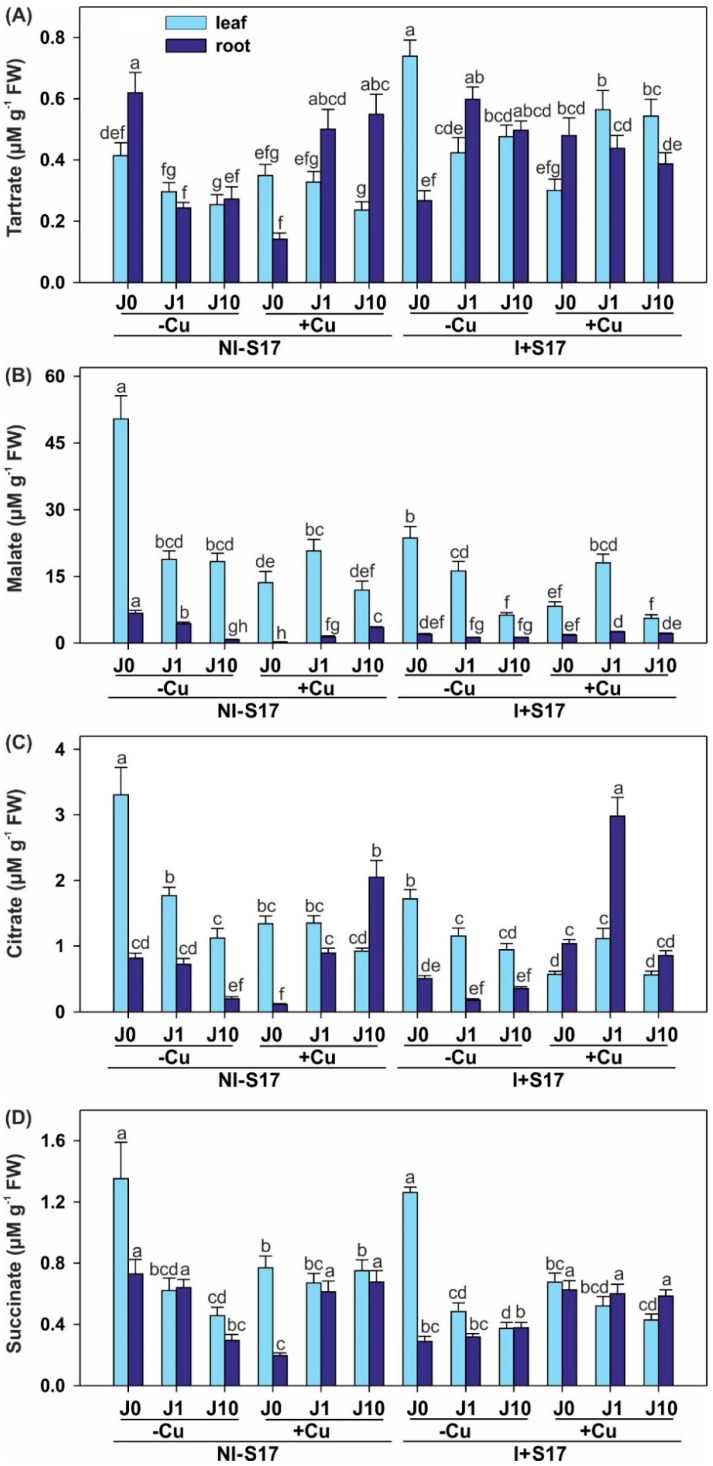
Concentration of low molecular weight organic acids in *Phaseolus coccineus* leaves and roots, as follows: tartrate (**A**); malate (**B**); citrate (**C**); and succinate (**D**). Data are means ± SD (*n* = 3). Values for the leaves and roots followed by the same letter are not significantly different (*p* < 0.05; Tukey’s test). Abbreviations: J0 = without methyl jasmonate (J); J1 = with 1 µM of J; J10 = with 10 µM of J; −Cu = without Cu; +Cu = with 50 µM Cu; NI − S17 = no inoculation with the S17 isolate; I + S17 = inoculation with the S17 isolate; FW = fresh weight.

In all of the treatments, the malate concentration was higher in the leaves than in the roots (Figure 8 and Table S3). Citrate and succinate were more intensively accumulated mostly in the leaves. The pattern of the tartrate accumulation was the least correlated with the part of the plant, with five treatments exhibiting a higher concentration in the leaves.

Regardless of the plant part, all of LMWOA levels in the NI − S17 variant with Cu addition decreased, except for the tartrate in the leaves, compared with the treatments without Cu excess. When J1 was applied to this treatment, an elevation of the tartrate content and a reduction of the malate levels were detected in the roots. After the J10 supplementation, there was an enhancement in all LMWOAs in the roots and in succinate in the leaves. In the I + S17 variant with Cu supplementation, the levels of all of LMWOAs decreased in the leaves and increased in the roots (except malate), compared with the treatments without Cu application. In the above treatment, J1 caused an elevation of the tartrate concentration in the leaves and a reduction in the roots, but J10 raised the LMWOAs level in the leaves and shortened the tartrate concentration in the roots.

The inoculation increased the tartrate and reduced the malate and citrate concentration in the leaves, and minimized the tartrate, malate, and succinate levels in the roots, compared with NI − S17. The J1 addition elevated the tartrate and lowered the citrate contents in the leaves. It also raised the tartrate and depleted the other LMWOAs levels in the roots. The supplementation with J10 increased the tartrate concentration in the leaves and roots, and reduced the malate content in the leaves. 

Under the inoculation with Cu treatment, the malate and citrate levels were reduced in the leaves, and the content of all of LMWOAs was stimulated compared with NI − S17. The J1 addition increased the tartrate level in the leaves and the malate and citrate (3.3-fold) contents in the roots. After the J10 application, higher tartrate and lower succinate levels were determined in the leaves, whereas reduced tartrate, malate, and citrate contents were shown in the roots. 

### 2.10. Relationship among the Studied Parameters 

**Figure 9:**
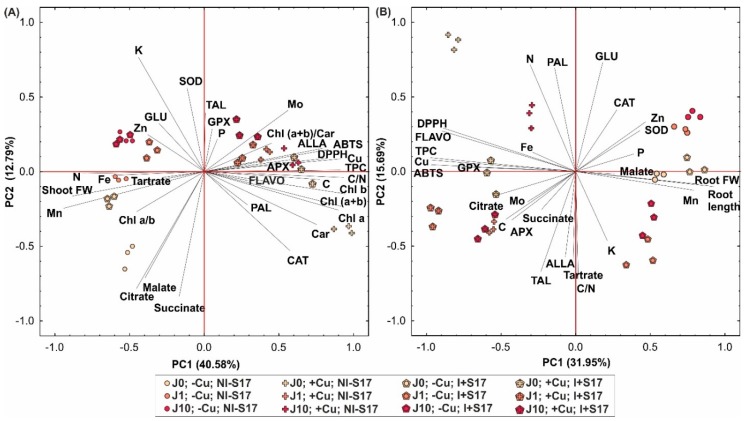
Scatter plot of the principal component analysis (PCA) for *P.* *coccineus* leaves/shoots (**A**) and roots (**B**) for the first and second axes for physiological parameters, for example, shoot/root fresh weight (FW), root length, Chl *a*/*b* ratio, concentration of chlorophyll a (Chl *a*), Chl *b*, carotenoids (Car), Chl (*a + b*), C, N, C/N, P, K, Fe, Mn, Mo, Zn, Cu, flavonoids (FLAVO), phenolics (TPC), allantoin (ALLA), tartrate, malate, citrate, and succinate; activity of SOD, CAT, APX, GPX, PAL, TAL, and GLU; and antioxidant potential (ABTS and DPPH tests). Abbreviations: J0 = without methyl jasmonate (J); J1 = with 1 µM of J; J10 = with 10 µM of J; −Cu = without Cu; +Cu = with 50 µM Cu; NI − S17 = no inoculation with the S17 isolate; I + S17 = inoculation with the S17 isolate.

The principal component analysis (PCA) based on the parameters analyzed in the *Phaseolus coccineus* leaves/shoots and roots showed a strong separation of the individuals into two groups, that is, Cu-treated and non-Cu-treated plants (Figure 9A,B). The variables with Cu were clearly correlated with axis 1 as follows: positively in the leaves and negatively in the roots. Moreover, the second principal component (PC) divided the leaf results into the following two groups: J-treated and non-J-treated. 

The first two components of the PCA analysis explained 53.37% of the total variance for the leaf parameters (Figure 9A) and 47.64% for the root parameters (Figure 9B). In the case of the leaves, the first component was positively loaded mainly by the concentration of Chls, TPC, and Cu, as well as the C/N ratio and antioxidant capacity, and negatively loaded by the concentration of Mn, Fe, and N and the shoot biomass (Figure 9A). The second component was determined positively mainly by the K concentration and SOD, and negatively by succinate, citrate, and malate. 

In the case of the roots, the first component was correlated positively mostly with the root length, its biomass, and Mn concentration, and negatively with the antioxidant capacity and the concentration of TPC, Cu, and FLAVO (Figure 9B). The second PC was determined positively primarily by the activity of GLU and PAL and the N concentration, and negatively by the C/N ratio, TAL activity, and the concentration of K, tartrate, and ALLA.

**Figure 10:**
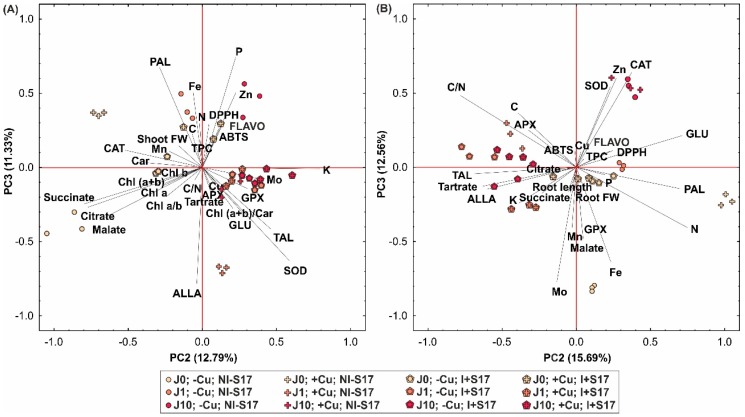
Scatter plot of the PCA for the *P. coccineus* leaves/shoots (**A**) and roots (**B**) for the second and third axes for physiological parameters, for example, shoot/root fresh weight (FW), root length, Chl *a*/*b* ratio, concentration of chlorophyll a (Chl *a*), Chl *b*, carotenoids (Car), Chl (*a + b*), C, N, C/N, P, K, Fe, Mn, Mo, Zn, Cu, flavonoids (FLAVO), phenolics (TPC), allantoin (ALLA), tartrate, malate, citrate, succinate; activity of SOD, CAT, APX, GPX, PAL, TAL, and GLU; and antioxidant potential (ABTS and DPPH tests). Abbreviations: J0 = without methyl jasmonate (J); J1 = with 1 µM of J; J10 = with 10 µM of J; −Cu = without Cu; +Cu = with 50 µM Cu; NI − S17 = no inoculation with the S17 isolate; I + S17 = inoculation with the S17 isolate.

The PCA based on the second and third PCs in the case of *Phaseolus coccineus* leaves/shoots and roots demonstrated a separation between the inoculated and non-inoculated plants (Figure 10A,B). The variables with the inoculation were positively correlated with both axes in the case of the leaves, and negatively correlated in the case of the roots. Furthermore, no-inoculation with S17 isolate in the leaves and roots were better separated from each other, as they were located mainly at the edge of the diagram. The treatments with the inoculation were generally located closer to the center of the diagram.

The second and third components of the PCA analysis explained 24.12% of the total variance for the leaf parameters (Figure 10A) and 28.25% for the root parameters (Figure 10B). In the case of the leaves, the second component was loaded positively chiefly by the K concentration; partially by the SOD and TAL activities; and negatively by succinate, citrate, and malate concentrations. The third PC was correlated positively mainly with the P, Fe, and Zn concentrations, as well as the PAL activity; negatively with the ALLA concentration; and partially with the SOD and TAL activities. 

In the case of the roots, the second PC was loaded positively mostly by the N concentration and GLU and PAL activity, and negatively by the C/N ratio, tartrate, and ALLA concentrations, and TAL activity. The third component was correlated positively predominantly with the CAT and SOD activities and Zn concentration, and negatively with the concentrations of Mo and Fe. 

## 3. Discussion

The interplay between J signaling and metal stress is important from the physiological point of view, but the correlation of these agents with the application of plant growth promoting bacteria seems to be promising for sustainable agriculture, as it can effectively stimulate and protect plant development, even in an adverse environment. Differently induced mechanisms of antioxidant production (enzymes and non-enzymatic compounds) may be the plausible cause of the varied responses. 

The PCA analysis confirmed clearly three groups of differences. First, there were differences between the Cu-treated and non-Cu-treated *Phaseolus coccineus* plants. Second, the results from the leaves discriminated between the J-treated and non-J-treated plants. Third, the leaves/shoots and roots showed differences between the inoculated and non-inoculated plants, with more pronounced disparities among the non-inoculated treatments. 

Under S17 inoculation, the plant biomass and root length did not change substantially, which means that the isolates with PGPB traits prepare the plant for better stress endurance in terms of biochemistry rather than via morphological features. Irrespective of the presence of J, Cu reduced the plant biomass, which is an evident effect of the metal excess [5]. The content of fluorescence pigments increased under the Cu stress, which is an earlier known phenomenon [5], and was quite stable and insensitive to the changes caused by inoculation. A possible explanation for the stable levels of Chls and Car could be their role as strong protectants of photosynthesis, which is a crucial process for plant survival.

The function of J results in the accumulation of H_2_O_2_, which serves as a secondary messenger in the induction of defense and resistance against biotic and abiotic stresses [12,37]. The literature data proved that a low CAT activity concurred with an elevated amount of toxic H_2_O_2_, but the high activity of the enzyme resulted in a reduction of the H_2_O_2_ level. The high activity of the peroxidase was correlated with the huge production of H_2_O_2_ [38]. Both compounds can participate in strengthening the cell wall. Some of our results confirmed the combined action, especially between SOD and CAT, not only in Cu excess, but also after inoculation, which is in line with other data [38]. The activities of antioxidant enzymes such as SOD, CAT, and peroxidases increased in *Pseudomonas* and *Bacillus* inoculated plants, which is partially in agreement with our findings, but our results suggested a dependency on the presence and concentration of J [3,12]. Similarly, as major defensive enzymes, SOD, CAT, and GPX showed significantly increased activity after bacterial infection, suggesting intensive use of overgenerated H_2_O_2_ in the oxidation of phenolics, which can be inhibitors of bacterial and fungal growth [3,12,38]. We proved that the S17 inoculation, both with and without Cu stress, induced plant alertness to the upcoming stress, and that not all of the enzymes measured showed the same tendency. Diversified enzyme activities supplement each other in the final protective effect on plants. Moreover, the enzyme activity depends on the length of the exposure time and changes during plant lifetime [5]. 

Evidence for the involvement of purine catabolism in stress protection in plants has recently emerged [15,21,39,40], with ALLA as an activator of JA/J metabolism, signaling, and responses involving abscisic acid (ABA) [15,41]. ALLA mitigates plant stress through decreasing ROS accumulation, directly as a ROS protectant [42], or indirectly [43]. On the other hand, it is still not clear whether the stimulatory effect of ALLA on plant growth in adverse conditions is related to its regulatory or signaling function or to its participation in metabolic processes (e.g., ALLA can serve as an alternative N source in the absence of its primary source) [41,44]. An elevated ALLA level during stress is essential for the development of plant tolerance [45], which was also demonstrated in our research. Endogenous ALLA can be exuded from roots into the soil, where it is readily absorbed causing a stimulatory effect on associated weeds and microorganisms [44]. 

Furthermore, at early stages of seedling growth, ALLA protects plants, which implies its developmental stage-specific function in seed germination [41]. Probably, ALLA prepares seedlings for potential future stress, minimizing the oxidative damage caused by superoxide accumulation [41]. 

We observed a higher ALLA concentration in the leaves than the roots of *P. coccineus* plants, which is in agreement with other findings for the two genotypes of *Phaseolus vulgaris* grown in stress conditions [46], or *Echium vulgare* from natural conditions and soil experiments with heavy metal pollution [40]. These data mostly agreed with our results obtained at the Cu excess after inoculation. Similar to our results, Díaz-Leal et al. [17] demonstrated an elevated ALLA concentration in the control plants, but after a long period (i.e., not earlier than after 35 days of *Phaseolus vulgaris* growth). In contrast to Wang et al. [44], we observed higher levels of ALLA in the leaves of the hydroponically cultured plants than in the roots. 

In *P. coccineus*, inoculation with S17 reduced the ALLA concentration, which rose after the J application. This indicates that J plays a vital role in plant readiness to stress conditions, and to the development of plant tolerance by elevating the ALLA level. Furthermore, the S17 inoculation in the Cu excess produced a J-dependent effect, compared with treatments of NI − S17. Therefore, the ALLA enhancement was predominantly associated with the presence of J, but not with the Cu application per se, although some published data show an ALLA elevation under heavy metal stress in *Echium vulgare* [21,40]. Moreover, exogenously applied 100 and 1000 µM ALLA can alleviate the negative impact of Cd on *Cucumis sativus* plants [39], and ALLA degradation is Mn^2+^-dependent [47].

Increases in PAL activity, a key enzyme in the phenylpropanoid pathway, are often considered as indicators of resistance [11]. PAL is a link between primary (protein synthesis) and an array of SM. In our experiment, the highest values of PAL were detected in the leaves of the Cu-treated, and then J10- and J1-supplemented plants. In the leaves of the I + S17, Cu-supplemented, and J1-treated plants, the PAL activity increased 3.3-fold. In contrast to the literature data that the deficiency of N [48] or P [49] increases PAL levels, we did not observe such an obvious relationship in our research. Similarly, our experiment did not clearly prove that the decrease in phenolics was related to a decrease in the total PAL activity [50]. Such discrepancies can be explained as follows: it is not only PAL activity that can give diversified effects on the accumulation of products derived from the phenylopropanoid pathway, but the activities of other enzymes present in the metabolic reaction chain may also react dissimilarly. 

In our research, the levels of TAL activity were lower than those of PAL, which agrees with the findings reported by Khan et al. [11]. Moreover, we proved that these activities mainly complemented each other (i.e., the higher activity of PAL corresponded with the lower activity of TAL, and vice versa). The products of PAL and TAL are a source of precursors of SM (e.g., FLAVO) [11], which was partially confirmed by our data. The finding that the total contents of the soluble phenols and flavonoids, as well as the specific activity of PAL and GLU were boosted after pathogen attack indicated a strengthening of the plant immune system against the intruder [3].

An accumulation of PR proteins, such as GLU, is stimulated by J [37], which was mainly proved by our experiments with inoculated and non-inoculated treatments. Generally, we showed a lower GLU activity after inoculation with a non-pathogenic bacterial isolate, which is in line with the finding that its enhanced activity resulted in intensified resistance against pathogens [51]. 

The enhancement of enzymatic and ABTS- or DPPH-scavenging activities at Cu excess can be explained by the elevated oxidative damage occurring before the plant is able to establish an active balance between the production and elimination of ROS, which is in agreement with the findings of the investigations conducted in stressful conditions [52]. The most significant changes in the antioxidant capacity were observed in the present study at combined application of two agents (i.e., S17 isolate with Cu). The elevation of the antioxidant capacity under Cu stress with inoculation showed different tendencies dependent mainly on the J concentration. Without J and with J10, the results were similar, and showed a reduction in ABTS- or DPPH-scavenging activities, whereas these activities were elevated at J1. The results showed that the bacterial isolates in the Cu-deprived environment helped the runner bean in its protection from the cell oxidation process through the increased activity of the antioxidant enzymes, which is in line with the data presented by Khalvandi et al. [52] for *Piriformospora indica* and *Mentha piperita*. At metal excess, a slightly more complicated tendency was noted. The inoculation with S17 caused a reduction of the antioxidant enzyme activity, but with a partial elevation of peroxidases. The high activity of the enzymes is probably a result of the detoxification of ROS in the plant tissues, which alleviates the negative effects of stress [52]. The inhibition of enzymatic activity can be associated with a high level of ROS, which is responsible for the generation of oxidative damage. Regardless of the plant part, without both inoculation and Cu, J evoked a higher activity of SOD. Basically, plants with higher levels of FLAVO and TPC had a higher anti-ROS activity, which is related to the high activity of hydrogen or electron donors [52].

The highest concentration of P was determined in the leaves and roots of the plants inoculated with S17. In contrast to our research, some scientists found a correlation between an increase in the phenolic compounds and P absorption [53]. The most significant stimulatory effect on TPC and FLAVO was exerted by the presence of Cu and inoculation, but not by the J level, as demonstrated by Khalvandi et al. [52]. The TPC and FLAVO accumulation may be caused by the intensification of the phenylpropanoid pathway and the induction of the activity of PAL [54]. 

We detected generally higher LMWOA concentrations in the leaves than the roots, with the exception of tartrate [55]. Thus, the tendency was analogical in the case of malate, citrate, and succinate, which stays in line with earlier statements [21,39]. 

Our research showed clear tendencies of LMWOA accumulation or reduction under different experimental conditions. Regardless of the plant part, the levels of all four organic acids were restricted under the J supplementation in the non-Cu-treated plants, mainly with lower LMWOA amounts in the inoculated treatments than in the non-inoculated ones. This may mean that inoculation hinders the synthesis of organic acids, or consumes greater amounts thereof. At the inoculation and presence of Cu, the J concentration diversified the content of malate in the leaves and the level of citrate in the roots. An opposite tendency was found in the non-inoculated Cu-treated plants; therefore, higher amounts or the synthesis of LMWOAs in the roots can accelerate Cu detoxification, in contrast to the data presented for Cd [20]. The inoculation elevated the LMWOA concentration, but this was reversed at the highest J dose. At the metal excess, the S17 effect was independent of the J level. Moreover, our results showed a higher concentration of malate than citrate. This can be associated with the lower accumulation of Cu in the plant tissues, compared with the reverse proportions of acids. As malate and citrate possess two and three carboxylic groups, respectively, they can bind a minimum of two or three ions of metal, respectively [20]. 

LMWOAs may be crucial for achieving an appropriate concentration of metals and non-metals in plant tissues by their involvement in the sequestration or translocation of elements [56]. Much more evidence is published for the contribution of LMWOAs in increasing metal tolerance [21,39,40] than their role in inoculation [57] or in the environment of J [5]. LMWOAs can be produced in high quantities by plants, especially in roots and seeds, but also in low quantities by soil microorganisms, which means that these acids, as root exudates, are involved in plant–microorganism interactions [58]. This interaction may be based on LMWOAs as signals for microorganism recognition or as precursors of phytohormone production. Organic acids can enhance microbial activity because soil microbiome mineralizes them quickly. We observed that the lower LMWOA concentrations and PAL and TAL activities in the roots were complemented with the higher activity of the antioxidant enzymes and GLU. However, in contrast to our results, some scientists reported positive correlations between the total LMWOA concentration; root activity; root-to-shoot ratio; and N, P, and K concentrations in roots [59]. Malate seems to be especially important because of its multifunctional roles. In *Miscanthus sacchariflorus*, exogenously applied malate alleviates Cd phytotoxicity by restraining ROS accumulation in an enzymatic and non-enzymatic way [60]. The amounts of LMWOAs can differ among species, for example, a higher citrate content was detected in *Vitis vinifera* and a higher malate level was determined in *V. amurensis* during cold acclimation [61]. LMWOAs have the capacity to complex copper in solutions, and their addition was found to elevate Cu accumulation in shoots [16].

Biofertilizers are mixtures containing microorganisms as active compounds with an ability to promote plant growth by increasing nutrient availability [62]. As an important macronutrient for plant growth and development, P is the key element for the synthesis of ATP, nucleic acids, and phospholipids; thus, a decrease in its content will restrict plant development [1]. We have demonstrated that the S17 isolate can substantially enhance the P uptake in the leaves and roots of non-Cu or J-treated *P. coccineus*, which suggests that this isolate has potential in PGP. Moreover, this treatment was also characterized by the highest N value for the leaves, high root length, and high activities of SOD, CAT, and APX in the roots. Similar results for P and N accumulation with the elevation of the enzymatic activity were obtained in investigations of chickpea inoculated with *Pseudomonas putida* [62]. An enhanced P uptake by plants can also be achieved via hormonal stimulation, mainly by IAA causing root growth, branching, and root hair development [63]. Furthermore, the P demand in the leaves of the control plants resulted in the elevation of citrate levels, which is in agreement with other reports [64].

Some of the many elements tested by us were characterized by the spectacular enhancement or reduction in inoculated plants. The effects seem to be related to the presence of J and Cu. Potassium is a coenzyme of various enzyme precursors, and its deficiency has been associated with the elevated activity of antioxidant enzymes [1]. Our research did not prove a clear dependence between the K level and enzyme activity or Car biosynthesis [1]. Fe catalyzes the reactions of the electron transfer and is intrinsic for antioxidant enzyme activity [65]. In the inoculated plants, we observed two reverse effects on the Fe level—a reduction in the treatments without Cu and elevation under the Cu stress. Mn participates in the activation of enzymes involved in N metabolism, RNA, and fatty acid biosynthesis [66]. Without Cu excess, we observed a significant rise in the Mn level in the *P. coccineus* roots after the application of the isolate. Mo is a component of enzymes and takes part in C and N cycles [67]. In our experiment, there was an elevated Mo level in the leaves after inoculation in the presence of J and Cu. At a high concentration, Mo can induce the production of SM [68], which is partially shown in our results. The level of Zn in the roots of the NI − S17 plants was enhanced by the J concentration. In turn, a reduced Zn concentration was detected after the inoculation. Zn is a cofactor of six enzyme groups involved in photosynthesis and hormone biosynthesis [69]. We demonstrated that the inoculation had no influence on the Cu accumulation in plant tissues. 

The results presented here provide evidence that Cu alleviates plant stress, J modulates plant response, and the bacterial isolate has the potential to affect the homeostasis of the plants and interplay in stress signaling. The present analyses of various metabolites and elements measured in our experiment, as well as the PCA results, demonstrated the potential of isolate S17 in the stimulation of plant well-being status in control and stress conditions. Therefore, its plant growth promoting abilities are worth deeper exploitation in the future. 

## 4. Materials and Methods

### 4.1. S17 Isolate Activity

In our previous study [35], the S17 isolate was biochemically identified using API 20 NE V8.0 tests, and was characterized by ACC (1-aminocyclopropane-1-carboxylate) deaminase activity, MIC for Cu, and the ability to synthesize IAA and produce siderophores (on the chrome azurol S agar medium). A germination test of *Phaseolus coccineus* plants inoculated with the S17 isolate was carried out as well [35].

The capability of the biofilm formation on polystyrene 96-well microtitre multi-well plates was determined according to O’Toole and Kolter [70], and Hossain and Tsuyumu [71]. The effect of Cu on the growth and activity of the S17 isolate was studied in cultures with Cu (50 µM) and without metal addition. The ability of the S17 isolate to synthesize IAA, phenolic and Fe(III) complexing compounds, and hydroxamate and catechole siderophores was determined in supernatants of the liquid culture obtained after 24- and 48-h of incubation at 20 °C. Three different media supplemented with Cu and 500 µg tryptophan (Trp) (i.e., an IAA precursor), were used, namely: (1) minimal medium (MM9) with 0.4% glucose; (2) MM9 with 0.4% sucrose; and Luria–Bertani broth (LB) containing 0.5% yeast extract, 1% trypton, 1% NaCl. 

The concentration of IAA was measured as described in Hanaka et al. [35]. The phenolic compounds were determined by the modified Folin–Ciocalteau method [72]. The amount of Fe(III)-chelating compounds produced by the S17 isolate was measured in the reaction with FeCl_3_, with the use of desferrioxamine B (DFOB), according to modified methods of Gibson and Magrath [73], and Atkin et al. [74]. Hydroxamate siderophores were determined using the modified Csaky [75], and Gibson and Magrath [73] method with NH_2_OH-HCl. The concentration of the catechole siderophores was determined using the modified Arnow [76] method with 3,4-dihydroxybenzoic acid. 

For further calculations, the protein concentration was quantified with the Bradford method [77].

### 4.2. Plant Material, Growth Condition, and Experimental Design 

In the experiment, three-day old runner bean seedlings (*Phaseolus coccineus* L. cv. Piękny Jaś) were grown hydroponically in pots (five plants per pot) filled with 1.5 L of Hoagland nutrient solution for 14 days with continuous aeration. The evaporated solution was supplemented with dH_2_O. The plants were cultivated in a growth chamber in the following conditions: 22/18 °C (day/night), 16-h photoperiod, and photosynthetic photon flux density of 120 mmol·m^−2^·s^−1^. After one day of hydroponic culture, the nutrient solution was supplemented with the bacterial isolate at a concentration of 1 × 10^8^ colony-forming units (CFU). The isolate was biochemically identified (API test) as *Pseudomonas luteola* derived from Spitsbergen soil (N 77°33′55′′ E 14°28′57′′) [35], and designated as S17. After two days of hydroponic culture, MJ (95%) was added at concentrations of 1 or 10 µM (J1 or J10, respectively). After three days of hydroponic culture, Cu was used as CuSO_4_·5H_2_O at a concentration of 50 µM. Finally, 12 treatments were analyzed without and with the inoculation of *P. luteola*, as follows: control, J1, J10, Cu, J1 + Cu, and J10 + Cu. Fresh plants were used for the biomass analysis. For further biochemical analysis, the leaf and root samples were frozen in liquid N and stored at −80 °C.

### 4.3. Measurement of Growth Parameters

The fresh plants were divided into shoots and roots, and were weighted. The root length was measured.

### 4.4. Quantification of Pigments

Chl *a*, Chl *b*, and total Car were extracted using 80% acetone, and their concentrations were determined spectrophotometrically (UV-160A Shimadzu, Tokyo, Japan), according to Wellburn [78], and were calculated based on Lichtenthaler and Buschmann [79].

### 4.5. Measurement of Selected Elements in Leaves and Roots

The plant samples (leaves and roots rinsed in distilled water) were dried at 105 °C to a constant weight. The C and N contents were determined using a LECO CNS elementary analyzer (LECO Truspec CN, St. Joseph, MI, USA). The accuracy of the determinations was tested against certified reference material (calibration organic matters ref. no. 502-082 Tobacco Leaves, LECO Corporation).

In order to determine the pseudototal (hereafter referred to as total) content of the metals (e.g., K, Fe, Mn, Zn, Mo, Na, and Cu), the plant samples were dissolved with aqua regia (ISO 11466). The trace elements were determined using the flame atomic absorption spectrometry (F-AAS) technique (Agilent 240 FS F-AAS, Santa Clara, CA, USA). P was extracted with aqua regia too, and was determined with the Molybdenum Blue Method [80] and UV-VIS spectrophotometry (Perkin-Elmer, Model Lambda 12, Waltham, MA, USA).

### 4.6. Quantification of Flavonoids, Phenolics, and Antioxidant Capacity

The analyses of FLAVO, TPC, and AC were performed in 80% methanolic extracts of the shoots and roots (extraction 1 g of fresh weight (FW)/2 mL of 80% MeOH). The concentration of FLAVO, expressed as mg of rutin equivalent per gram of FW, was determined based on the formation of a flavonol–aluminium complex [81]. The TPC content, expressed as mg of gallic acid equivalents per gram of FW of plants, was determined using the Folin–Ciocalteau reagent, as described previously [72,82]. The antioxidant capacities expressed as mg of Trolox equivalent per gram of FW were measured using free radicals: ABTS [83] and DPPH [40]. 

### 4.7. Quantification of Antioxidant Enzymes

Preparation of samples and measurements of enzyme activity were carried out according to García-Limones et al. [84], with some modifications. Then, 1 g of plant tissue was homogenized in 4 mL of 50 mM potassium phosphate buffer, pH 7.5, containing 1 mM ethylenediaminetetraacetic acid (EDTA), 1 mM phenylmethanesulfonyl fluoride (PMSF), 5% (*w*/*v*) PVPP at 4 °C. The homogenate was centrifuged at 17,000× *g* for 20 min at 4 °C, and the supernatant was frozen in liquid N and stored at −80 °C for further analysis. The enzyme activity was measured spectrophotometrically at 25 °C in a final volume of 1 mL.

The reaction mixture for the determination of SOD activity consisted of a 50 mM potassium phosphate buffer, pH 7.8, 0.1 mM EDTA, 13 mM methionine, 75 µM nitroblue tetrazolium (NBT), 2 µM riboflavin, and different volumes of enzyme extract (between 10, 15, and 20 µL). The reaction started after the application of riboflavin, and, after 12-min incubation at room temperature under light, the inhibition of the photochemical reduction of NBT was measured as a decrease in absorbance in A_560_.

The reaction medium for the determination of CAT activity consisted of 50 mM potassium phosphate buffer, pH 7.0, 20 mM H_2_O_2_, and 20 µl of enzyme extract. The reaction started after the addition of H_2_O_2_. The H_2_O_2_ breakdown caused a decrease in A_240_.

The reaction mixture for the determination of APX activity consisted of 50 mM of potassium phosphate buffer, pH 7.0, 0.25 mM sodium ascorbate, 5 mM H_2_O_2_, and 50 µL of enzyme extract. The reaction started after the addition of H_2_O_2_. The oxidation of ascorbate was determined as a decrease in A_290_.

The reaction mixture for the determination of GPX activity consisted of 100 mM potassium phosphate buffer, pH 6.5, 15 mM guaiacol, 0.05 (*v*/*v*) H_2_O_2_, and 10 µl of differently diluted enzyme extract. The reaction started after the addition of H_2_O_2_. The oxidation of guaiacol was determined as an increase in A_470_.

Proteins were assayed according to Bradford [77].

### 4.8. Quantification of Defence-Related Enzymes

PAL and TAL were measured according to Patel et al. [85] and Khan et al. [11]. The reaction mixture contained 0.1 mL of enzyme extract, 0.65 mL of distilled water, and 0.5 mL of 0.1 M phenylalanine/tyrosine. The reaction mixture was incubated at 32 °C for 60 min. After the incubation, 0.5 mL of 5 N HCl was added so as to stop the reaction. PAL and TAL activities were measured at 290 and 320 nm following formation of trans-cinnamic acid and coumaric acid, respectively. 

GLU was assayed according to Alfonso et al. [86], and was measured with the method proposed by Nelson [87] and Somogyi [88], modified by Hope and Burns [89]. The reaction mixture contained 0.5 mL of a solution of the appropriate crude enzyme and 0.5 mL of laminarine from *Laminaria digitata* in phosphate buffer (pH 5.6). The reaction mixture was incubated at 37 °C for 12 h with gentle agitation (80 rpm). After the incubation, the absorbance was measured at 520 nm.

### 4.9. Quantification of ALLA and LMWOAs

An Agilent 7100 Capillary Electrohoresis set coupled with a diode-array detector (UV-Vis/DAD, 190–600 nm; Agilent Technologies) was used for the separation and determination of ALLA and LMWOAs. 

The analysis of ALLA was conducted according to Dresler et al. [90]. The analysis of ALLA was carried out in a 50 µm i.d. silica capillary with a total length of 64.5 cm, filled with electrolytes consisting of a 50 mM borate solution, pH 9.2. ALLA was identified at λ = 192 nm based on comparison of the retention time and absorption spectrum similarity in a 190–400 nm range with allantoin standards purchased in Sigma-Aldrich (St. Louis, MO, USA).

The LMWOAs were prepared and measured as described in Hanaka et al. [6]. 

### 4.10. Statistical Analysis

The data were presented as the mean value with standard deviation (SD), and the statistical analyses were conducted using Statistica 12.5 (Stat Soft. Inc., Cracov, Poland). The data were subjected to an analysis of variance (three-way ANOVA) with a post-hoc Tukey’s test at *p* < 0.05. The principal component analysis (PCA) was carried out to determine the relationship among the variables studied. The PCA analyses were based on three values of variables for a given treatment, but each of the three values for the biomass data was the mean from the five plants. The completely randomized experiments involved 12 treatments (two bacterial isolates × two Cu × three J treatments) with three pots and five plants per pot (15 plants per treatment). The whole experiment was independently repeated two times under the same growth conditions. The separately collected roots and shoots from one pot (five plants) were mixed and divided into samples for the analysis of all variables. All of the analyses were performed in the three independent biological replicates (*n* = 3). 

## 5. Conclusions

The results evidenced that the inoculation with the S17 isolate originating from Spitsbergen soil mitigated the effect of Cu stress—it improved plant growth and had a significant effect on the resistance of the plant to the metal. Under the Cu stress inoculation with S17, the isolate caused the elevation of GPX and a reduction of SOD and CAT activities, and a significant increase of FLAVO and TPC in the leaves. In the presence of J10 and the S17 isolate, an elevation of the shoot FW; K concentration; TAL activity; and FLAVO, TPC, and AC levels in the leaves and the APX and GPX (also at J1) activity in the roots was observed. In the absence of Cu, the S17 isolate contributed to enhancement of N, P, and Mn uptake and increased the root length and the shoot-to-root ratio. The effects of inoculation were associated with the application of Cu and J, which modified the plant response (e.g., the PAL, TAL, and LMWOAs levels), mainly in a J concentration-dependent manner. The results presented here provide evidence that Cu alleviates plant stress, J modulates plant response, and the bacterial isolate S17 has not only the potential to plant growth promotion but also influence on plant homeostasis. This interplay of the isolate in stress signaling has fundamental importance for determining plant–soil–metal associations. 

## Figures and Tables

**Table 1:** Phenotype characteristics of the S17 isolate on different media, and C sources with and without the addition of Cu and the indoleacetic acid (IAA) precursor—tryptophan (Trp). Data are means ± SD (*n* = 3). Abbreviations: MM9 = minimal medium; LB = Luria–Bertani broth; Trp−Cu = medium with Trp and without Cu; −Cu = medium without Cu; +Cu = medium with 50 µM Cu; Fe(III)CC = Fe(III) complexing compounds; CAS = chrome azurol S blue medium; psu = percentage siderophore unit; nd = not detected.

Parameter/ Metabolite	Incubation Time	Medium/Treatment								
		MM9 Glucose			MM9 Sucrose			LB		
		+Trp −Cu	−Trp −Cu	−Trp +Cu	+Trp −Cu	−Trp −Cu	−Trp +Cu	+Trp −Cu	−Trp −Cu	−Trp +Cu
Growth (OD_600_)	24 h	0.037 ± 0.007	0.027 ± 0.006	0.006 ± 0.001	0.028 ± 0.001	0.012 ± 0.004	0.001 ± 0.001	1.221 ± 0.011	1.229 ± 0.001	1.282 ± 0.026
	48 h	0.095 ± 0.001	0.101 ± 0.002	0.054 ± 0.001	0.084 ± 0.007	0.079 ± 0.006	0.004 ± 0.003	1.274 ± 0.029	1.252 ± 0.023	1.299 ± 0.002
Proteins (µg·mL^−1^)	24 h	5.79 ± 1.46	1.94 ± 1.14	4.87 ± 1.56	3.10 ± 1.60	2.58 ± 1.65	6.69 ± 1.75	8.62 ± 2.87	3.65 ± 1.46	5.24 ± 1.26
	48 h	7.56 ± 1.48	3.29 ± 0.09	2.68 ± 0.31	2.41 ± 0.24	1.79 ± 0.04	2.12 ± 1.47	7.91 ± 0.21	12.31 ± 1.65	7.58 ± 1.29
pH	24 h	3.80 ± 0.05	3.90 ± 0.02	3.89 ± 0.03	3.74 ± 0.06	3.69 ± 0.09	3.82 ± 0.02	8.44 ± 0.15	8.54 ± 0.12	8.52 ± 0.10
	48 h	3.24 ± 0.01	3.21 ± 0.04	3.79 ± 0.01	3.70 ± 0.05	4.39 ± 0.03	3.69 ± 0.02	8.97 ± 0.14	8.92 ± 0.21	8.90 ± 0.13
IAA (µg·mL^−1^)	24 h	4.95 ± 0.02	0.00 ± 0.00	0.00 ± 0.00	6.50 ± 0.40	0.00 ± 0.00	0.12 ± 0.02	3.75 ± 0.19	1.53 ± 0.12	1.40 ± 0.07
	48 h	3.70 ± 0.03	0.43 ± 0.13	0.20 ± 0.02	2.60 ± 0.09	0.00 ± 0.00	0.00 ± 0.00	9.35 ± 0.28	3.70 ± 0.03	5.13 ± 0.02
Fe(III)CC FeCl_3_ test (µg·mL^−1^)	24 h	5.31 ± 0.11	5.05 ± 0.09	4.93 ± 0.01	6.41 ± 0.38	5.49 ± 0.05	4.93 ± 0.01	3.79 ± 0.20	4.03 ± 0.07	1.54 ± 0.12
	48 h	5.21 ± 0.01	6.23 ± 0.07	4.93 ± 0.06	5.07 ± 0.03	4.94 ± 0.18	4.76 ± 0.03	5.49 ± 0.50	6.90 ± 0.07	3.35 ± 0.19
Siderophores total CAS test (psu unit)	24 h	8.09 ± 0.08	3.47 ± 0.04	1.60 ± 0.08	15.01 ± 0.07	5.75 ± 0.01	0.49 ± 0.01	51.80 ± 0.02	51.01 ± 0.04	47.08 ± 0.02
	48 h	19.98 ± 0.02	15.92 ± 0.15	1.57 ± 0.08	20.80 ± 0.06	14.62 ± 0.03	0.89 ± 0.07	62.01 ± 0.02	71.10 ± 0.01	62.84 ± 0.03
Hydroxamate siderophores (µg·mL^−1^)	24 h	5.29 ± 0.84	2.46 ± 0.10	0.88 ± 0.07	1.81 ± 0.37	0.32 ± 0.06	0.21 ± 0.17	7.42 ± 0.64	0.35 ± 0.03	0.23 ± 0.05
	48 h	2.16 ± 0.24	4.29 ± 0.58	0.23 ± 0.03	2.37 ± 0.17	0.57 ± 0.03	1.50 ± 1.15	5.56 ± 0.32	1.64 ± 0.03	0.19 ± 0.01
Catechole siderophores (µg·mL^−1^)	24 h	1.21 ± 0.04	1.02 ± 0.01	1.09 ± 0.13	1.25 ± 0.19	1.05 ± 0.02	1.20 ± 0.17	1.15 ± 0.03	2.45 ± 0.12	1.19 ± 0.13
	48 h	1.05 ± 0.03	0.99 ± 0.10	0.98 ± 0.05	1.00 ± 0.04	0.92 ± 0.01	1.12 ± 0.03	0.93 ± 0.04	0.97 ± 0.17	1.19 ± 0.01
Phenolic compounds (µg·mL^−1^)	24 h	nd	25.04 ± 0.68	14.61 ± 0.20	nd	14.74 ± 0.76	16.32 ± 2.28	nd	nd	nd
	48 h	nd	20.18 ± 0.01	25.32 ± 1.71	nd	17.30 ± 1.37	30.80 ± 1.31	nd	nd	nd

**Table 2:** Total flavonoids (FLAVO), total phenolic compounds (TPC), and antioxidant capacity (AC) determined using ABTS (2-azino-bis-3-ethylbenzthiazoline-6-sulphonic acid) and DPPH (2,2-diphenyl-1-picrylhydrazyl) in the *Phaseolus coccineus* leaves and roots. Data are means ± SD (*n* = 3). Values in each column followed by the same letter are not significantly different (*p* < 0.05; Tukey’s test). Abbreviations: J0 = without methyl jasmonate (J); J1 = with 1 µM of J; J10 = with 10 µM of J; −Cu = without Cu; +Cu = with 50 µM Cu; NI − S17 = no inoculation with the S17 isolate; I + S17 = inoculation with the S17 isolate; FW = fresh weight.

Treatment			FLAVO		TPC		AC			
							ABTS		DPPH	
			mg·g^−1^·FW							
			Leaf	Root	Leaf	Root	Leaf	Root	Leaf	Root
J0	−Cu	NI − S17	0.22 ± 0.02 ^e,f^	0.012 ± 0.001 ^d^	0.72 ± 0.06 ^e,f^	0.25 ± 0.02 ^e,f^	0.33 ± 0.01 ^d^	0.025 ± 0.002 ^f^	0.074 ± 0.007 ^g,h^	0.006 ± 0.001 ^f^
J1	−Cu	NI − S17	0.22 ± 0.02 ^e,f^	0.016 ± 0.002 ^d^	0.57 ± 0.05 ^f,g^	0.26 ± 0.03 ^e,f^	0.30 ± 0.03 ^d^	0.033 ± 0.002 ^e,f^	0.061 ± 0.006 ^g,h^	0.003 ± 0.000 ^f^
J10	−Cu	NI − S17	0.29 ± 0.02 ^d^	0.012 ± 0.001 ^d^	0.91 ± 0.08 ^d,e^	0.22 ± 0.02 ^e,f^	0.61 ± 0.06 ^c^	0.026 ± 0.001 ^f^	0.203 ± 0.018 ^d,e^	0.004 ± 0.000 ^f^
J0	+Cu	NI − S17	0.40 ± 0.02 ^b,c^	0.055 ± 0.005 ^a^	1.52 ± 0.09 ^b^	0.75 ± 0.03 ^a^	0.78 ± 0.03 ^a,b^	0.133 ± 0.007 ^a^	0.305 ± 0.030 ^a,b^	0.093 ± 0.011 ^a^
J1	+Cu	NI − S17	0.24 ± 0.02 ^d,e^	0.038 ± 0.003 ^c^	1.16 ± 0.11 ^c^	0.66 ± 0.03 ^b,c^	0.56 ± 0.06 ^c^	0.116 ± 0.005 ^b^	0.114 ± 0.009 ^f,g^	0.059 ± 0.006 ^c,d^
J10	+Cu	NI − S17	0.36 ± 0.03 ^c^	0.054 ± 0.003 ^a^	1.44 ± 0.14 ^b^	0.67 ± 0.04 ^a,b,c^	0.55 ± 0.05 ^c^	0.120 ± 0.006 ^a,b^	0.338 ± 0.033 ^a^	0.077 ± 0.007 ^b^
J0	−Cu	I + S17	0.17 ± 0.02 ^f^	0.012 ± 0.000 ^d^	0.47 ± 0.05 ^g^	0.20 ± 0.01 ^f^	0.33 ± 0.02 ^d^	0.032 ± 0.003 ^e,f^	0.050 ± 0.005 ^h^	0.004 ± 0.000 ^f^
J1	−Cu	I + S17	0.24 ± 0.02 ^d,e^	0.015 ± 0.003 ^d^	0.66 ± 0.06 ^f,g^	0.29 ± 0.03 ^d,e^	0.35 ± 0.04 ^d^	0.041 ± 0.003 ^d,e^	0.088 ± 0.008 ^g,h^	0.008 ± 0.001 ^f^
J10	−Cu	I + S17	0.24 ± 0.02 ^d,e,f^	0.016 ± 0.002 ^d^	0.63 ± 0.05 ^f,g^	0.35 ± 0.03 ^d^	0.42 ± 0.02 ^d^	0.051 ± 0.005 ^d^	0.101 ± 0.011 ^f,g,h^	0.003 ± 0.000 ^f^
J0	+Cu	I + S17	0.56 ± 0.04 ^a^	0.055 ± 0.003 ^a^	1.77 ± 0.11 ^a^	0.66 ± 0.03 ^b,c^	0.81 ± 0.06 ^a^	0.103 ± 0.005 ^c^	0.273 ± 0.029 ^b,c^	0.030 ± 0.001 ^e^
J1	+Cu	I + S17	0.44 ± 0.02 ^b^	0.047 ± 0.002 ^b^	1.51 ± 0.13 ^b^	0.73 ± 0.06 ^a,b^	0.67 ± 0.06 ^b,c^	0.133 ± 0.005 ^a^	0.222 ± 0.010 ^c,d^	0.070 ± 0.003 ^b,c^
J10	+Cu	I + S17	0.36 ± 0.02 ^c^	0.032 ± 0.002 ^c^	0.96 ± 0.08 ^c,d^	0.59 ± 0.06 ^c^	0.63 ± 0.03 ^c^	0.131 ± 0.005 ^a^	0.153 ± 0.026 ^e,f^	0.051 ± 0.005 ^d^

## Supplementary Materials

Supplementary materials can be found at https://www.mdpi.com/1422-0067/20/8/1909/s1.

## Abbreviations

J	Methyl jasmonate
Chl *a*	Chlorophyll *a*
Chl *b*	Chlorophyll *b*
Car	Carotenoids
ABTS	2-Azino-bis-3-ethylbenzthiazoline-6-sulphonic acid
DPPH	2,2-Diphenyl-1-picrylhydrazyl
AC	Antioxidant capacity
FLAVO	Total flavonoids
TPC	Total phenolics compounds
SM	Secondary metabolites
SOD	Superoxide dismutase
CAT	Catalase
APX	Ascorbate peroxidase
GPX	Guaiacol peroxidase
PAL	Phenylalanine ammonia-lyase
TAL	Tyrosine ammonia-lyase
GLU	β-1,3-Glucanase
ALLA	Allantoin
LMWOAs	Low molecular weight organic acids
PGP	Plant growth promotion
