# Supplementary material for: Bacterial Isolate Inhabiting Spitsbergen Soil Modifies the Physiological Response of Phaseolus coccineus in Control Conditions and under Exogenous Application of Methyl Jasmonate and Copper Excess
Source: Int J Mol Sci. 2019 Apr 17;20(8):1909. doi: 10.3390/ijms20081909 (PMC6514558; doi:10.3390/ijms20081909)
Supplement: Supplementary file 1 [file ijms-20-01909-s001.pdf]

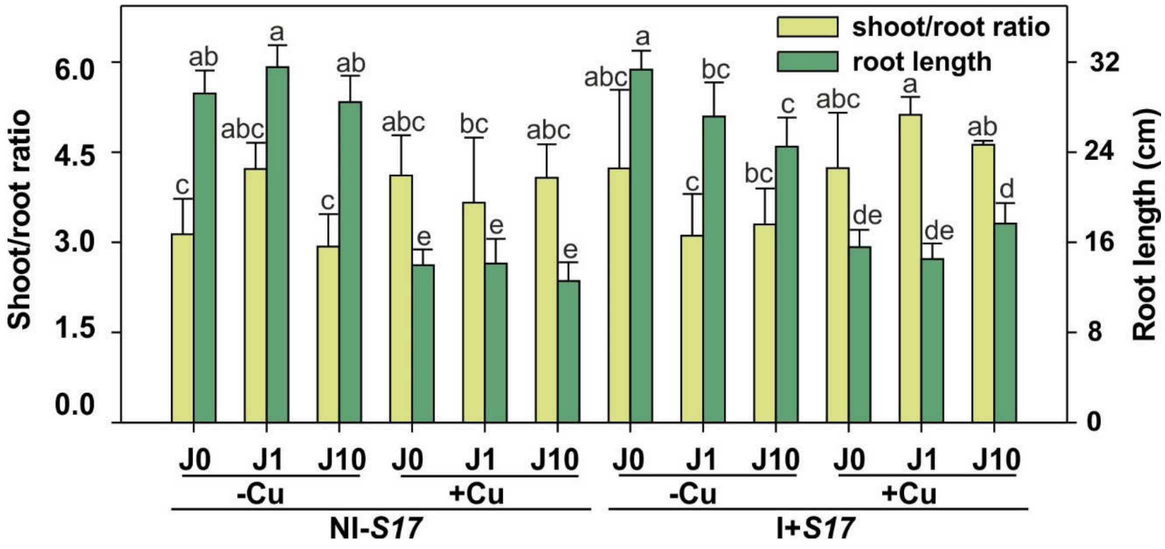

Supplementary Figure S1. Biometric parameters of *Phaseolus coccineus* shoots and roots: shoot-to-root ratio and root length. Data are means ± SD (n = 30). Values for shoots and roots followed by the same letter are not significantly different ($p < 0.05$; Tukey's test). Abbreviations: J0 = without methyl jasmonate (J), J1 = with 1 µM of J, J10 = with 10 µM of J; −Cu = without Cu, +Cu = with 50 µM Cu; NI − S17 = no inoculation with the S17 isolate, I + S17 = inoculation with the S17 isolate.

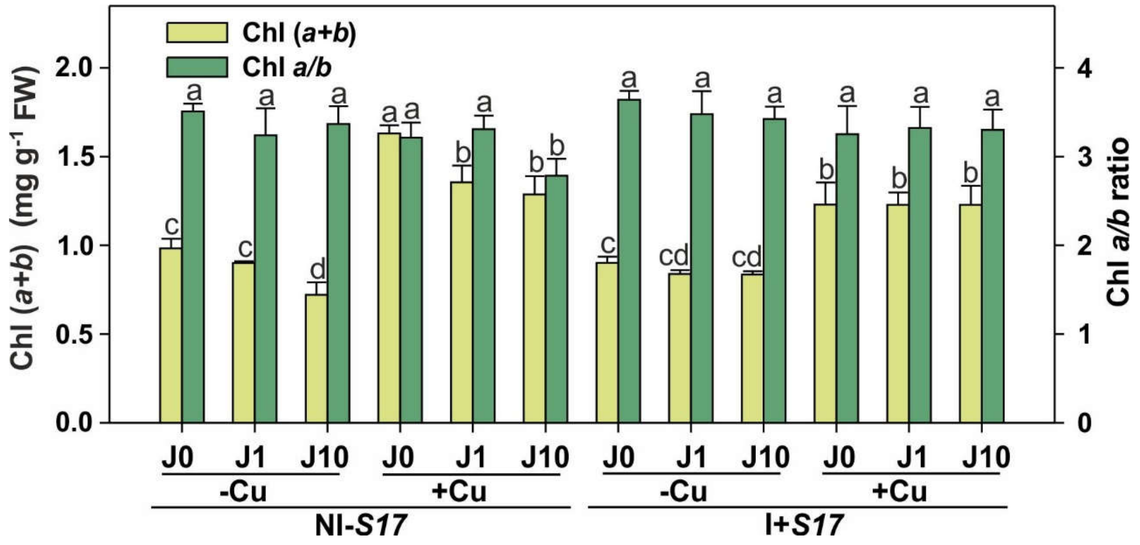

Supplementary Figure S2. Ratios of photosynthetic pigments in *Phaseolus coccineus* leaves: sum of chlorophylls (Chl *a*+*b*) and chlorophyll *a* to *b* ratio (Chl *a*/*b*). Data are means ± SD (n = 3). Values followed by the same letter are not significantly different ($p < 0.05$; Tukey's test). Abbreviations: J0 = without methyl jasmonate (J), J1 = with 1 µM of J, J10 = with 10 µM of J; −Cu = without Cu, +Cu = with 50 µM Cu; NI − S17 = no inoculation with the S17 isolate, I + S17 = inoculation with the S17 isolate, FW = fresh weight, Chl = chlorophyll.

Supplementary Table S1. Results of the multivariate analysis of variance by three-way ANOVA for biomass data, and concentration of photosynthetic pigments and elements. The variables were tested with the following factors: bacterial isolate S17 (S17), Cu, and methyl jasmonate (J). F-value with the level of significance ($p < 0.001$ – ***; $p < 0.01$ – **; $p < 0.05$ – *) were presented. Abbreviations: FW = fresh weight, Chl *a* = chlorophyll *a*, Chl *b* = chlorophyll *b*, Car = carotenoids.

Source of variation	Shoot FW		Root FW		Shoot/Root ratio		Root length		Chl *a*		Chl *b*		Car		Chl (*a*+*b*) /Car		Chl (*a*+*b*)		Chl *a*/*b*	
	F	p	F	p	F	p	F	p	F	p	F	p	F	p	F	p	F	p	F	p
S17	0.3	***	1.9	ns	6.3	*	0.1	ns	16.8	***	41.5	***	12.7	***	2.2	ns	25.5	***	9.8	**
Cu	235.0	***	294.8	***	24.3	***	1123.0	***	451.0	***	551.5	***	348.3	***	38.6	***	553.9	***	20.9	***
J	0.8	ns	1.9	ns	1.1	ns	5.9	**	23.7	***	9.1	***	77.2	***	19.1	***	22.6	***	4.1	*
S17×Cu	6.2	**	1.5	ns	3.2	ns	28.4	***	17.9	***	27.2	***	22.3	***	0.1	ns	23.3	***	0.2	ns
S17×J	5.8	*	3.4	*	0.6	ns	7.3	**	14.8	***	6.1	**	17.1	***	0.1	ns	14.2	***	1.3	ns
Cu×J	2.2	ns	0.5	ns	1.8	ns	10.0	***	0.4	***	6.0	**	12.3	***	16.2	***	1.3	ns	3.6	*
S17×Cu×J	9.2	***	2.0	ns	10.0	***	11.1	***	3.5	***	5.1	**	42.4	***	17.9	***	3.8	*	4.1	*

Source of variation	C				N				C/N ratio				K				P			
	Leaf		Root		Leaf		Root		Leaf		Root		Leaf		Root		Leaf		Root	
	F	p	F	p	F	p	F	p	F	p	F	p	F	p	F	p	F	p	F	p
S17	0.1	ns	4.3	*	5.5	*	145.6	***	5.4	*	65.2	***	62.7	***	0.5	ns	70.1	***	147.8	***
Cu	32.0	***	18.3	***	113.8	***	47.5	***	214.9	***	0.1	ns	26.4	***	5.1	*	2.2	ns	247.7	***
J	2.6	ns	8.5	**	0.0	ns	136.1	***	3.4	*	61.8	***	114.4	***	5.3	*	0.8	ns	317.2	***
S17×Cu	0.1	ns	9.1	**	0.1	ns	26.9	***	1.1	ns	0.1	ns	48.7	***	6.6	*	8.6	**	227.9	***
S17×J	0.8	ns	0.3	ns	5.3	*	81.9	***	3.2	ns	11.2	***	53.6	***	1.3	ns	80.0	***	473.5	***
Cu×J	3.0	ns	6.4	**	2.4	ns	29.6	***	8.8	**	8.1	**	10.0	***	16.8	***	67.1	***	132.0	***
S17×Cu×J	0.6	ns	9.1	**	10.8	***	81.4	***	3.3	ns	55.9	***	8.2	**	4.1	*	140.2	***	155.6	***

Source of variation	Fe				Mn				Zn				Mo				Cu			
	Leaf		Root		Leaf		Root		Leaf		Root		Leaf		Root		Leaf		Root	
	F	p	F	p	F	p	F	p	F	p	F	p	F	p	F	p	F	p	F	p
S17	109.0	***	1.9	ns	1.4	ns	142.7	***	53.6	***	272.6	***	53.4	***	33.1	***	0.5	ns	7.8	*
Cu	786.2	***	41.9	***	1644.8	***	2368.0	***	137.8	***	232.3	***	983.4	***	5.9	*	1435.4	***	5225.2	***
J	54.0	***	157.0	***	11.4	***	262.9	***	153.3	***	246.0	***	158.6	***	61.7	***	12.5	***	4.3	*
S17×Cu	58.2	***	173.4	***	16.6	***	63.4	***	225.5	***	127.7	***	15.7	***	18.7	***	6.8	*	5.0	*
S17×J	15.1	***	24.7	***	8.3	**	6.2	**	21.8	***	173.6	***	154.1	***	97.1	***	3.2	ns	0.0	ns
Cu×J	144.3	***	50.9	***	29.1	***	182.2	***	55.0	***	82.2	***	36.3	***	7.1	**	24.2	***	1.7	ns
S17×Cu×J	11.2	***	16.0	***	2.1	ns	6.1	***	192.0	***	51.9	***	241.4	***	3.7	*	5.2	***	0.3	ns

Supplementary Table S2 Results of the multivariate analysis of variance by three-way ANOVA for concentration of total flavonoids (FLAVO) and total phenolic compounds (TPC), antioxidant capacity, and enzymes activity. The variables were tested with the following factors: bacterial isolate S17 (S17), Cu, and methyl jasmonate (J). F-value with the level of significance ($p < 0.001$ – ***; $p < 0.01$ – **; $p < 0.05$ – *) were presented. Abbreviations: ABTS = 2-azino-bis-3-ethylbenzthiazoline-6-sulphonic acid, DPPH = 2,2-diphenyl-1-picrylhydrazyl, SOD = superoxide dismutase, CAT = catalase, APX = ascorbate peroxidase, GPX = guaiacol peroxidase, PAL = phenylalanine ammonia-lyase, TAL = tyrosine ammonia-lyase, GLU = β-1,3-glucanase.

Source of variation	FLAVO				TPC				ABTS				DPPH			
	Leaf		Root		Leaf		Root		Leaf		Root		Leaf		Root	
	F	p	F	p	F	p	F	p	F	p	F	p	F	p	F	p
S17	42,4	***	5,7	*	4,4	***	0,0	ns	0,7	ns	16,8	***	30,9	***	74,7	***
Cu	481,2	***	1590,3	***	757,7	***	1803,0	***	394,2	***	3268,7	***	478,9	***	1604,1	***
J	16,0	***	15,0	***	12,0	***	3,0	ns	17,5	***	13,7	***	53,2	***	0,7	ns
S17×Cu	90,3	***	9,9	**	12,9	***	13,0	***	19,2	***	23,1	***	0,1	ns	83,3	***
S17×J	26,4	***	21,4	***	41,9	***	14,9	***	7,8	**	34,3	***	94,0	***	64,0	***
Cu×J	65,4	***	38,5	***	44,9	***	14,2	***	61,8	***	0,2	ns	49,7	***	0,8	***
S17×Cu×J	8,5	**	43,6	***	14,3	***	13,9	***	6,8	**	18,8	***	14,3	***	44,6	***

Source of variation	SOD				CAT				APX				GPX			
	Leaf		Root		Leaf		Root		Leaf		Root		Leaf		Root	
	F	p	F	p	F	p	F	p	F	p	F	p	F	p	F	p
S17	23.8	***	322.5	***	59.4	***	56.3	***	46.3	***	255.3	***	17.9	***	216.2	***
Cu	5.5	*	145.5	***	321.7	***	33.2	***	127.8	***	895.9	***	0.1	ns	1014.7	***
J	412.4	***	72.0	***	250.2	***	166.7	***	34.3	***	94.2	***	4.9	*	135.4	***
S17×Cu	414.0	***	380.2	***	70.6	***	110.4	***	56.6	***	43.0	***	2.9	ns	104.6	***
S17×J	102.3	***	258.1	***	92.9	***	450.5	***	89.9	***	116.5	***	14.1	***	521.2	***
Cu×J	106.8	***	68.1	***	253.9	***	34.3	***	1.9	ns	399.0	***	0.5	ns	99.0	***
S17×Cu×J	178.6	***	43.0	***	31.1	***	133.8	***	18.8	***	594.0	***	0.7	ns	353.9	***

Source of variation	PAL				TAL				GLU			
	Leaf		Root		Leaf		Root		Leaf		Root	
	F	p	F	p	F	p	F	p	F	p	F	p
S17	315.2	***	34.4	***	40.1	***	65.9	***	1.5	ns	760.8	***
Cu	17.6	***	2.8	ns	0.7	ns	3.1	ns	14.1	***	5.8	*
J	32.2	***	27.7	***	24.4	***	19.6	***	125.2	***	6.6	**
S17×Cu	44.4	***	18.5	***	25.2	***	9.8	**	0.0	ns	3.3	ns
S17×J	96.2	***	0.4	ns	4.0	*	1.0	ns	93.4	***	50.5	***
Cu×J	386.7	***	46.7	***	20.8	***	19.4	***	89.6	***	4.9	*
S17×Cu×J	185.6	***	5.6	**	70.9	***	1.8	ns	1.5	ns	43.4	***

Supplementary Table S3. Results of the multivariate analysis of variance by three-way ANOVA for concentration of allantoin (ALLA) and low molecular weight organic acids (LMWOAs). The variables were tested with the following factors: bacterial isolate S17 (S17), Cu, and methyl jasmonate (J). F-value with the level of significance ($p < 0.001$ – ***; $p < 0.01$ – **; $p < 0.05$ – *) were presented.

Source of variation	ALLA				Tartrate				Malate				Citrate				Succinate			
	Leaf		Root		Leaf		Root		Leaf		Root		Leaf		Root		Leaf		Root	
	F	p	F	p	F	p	F	p	F	p	F	p	F	p	F	p	F	p	F	p
S17	82.8	***	340.5	***	186.6	***	13.9	***	135.3	***	156.9	***	133.1	***	20.5	***	23.8	***	10.7	***
Cu	152.9	***	27.9	***	10.9	**	0.0	***	134.6	***	94.7	***	162.8	***	440.3	***	16.7	***	35.2	***
J	86.3	***	328.4	***	9.1	**	7.1	***	95.9	***	30.7	***	81.4	***	66.5	***	114.8	***	7.4	**
S17×Cu	8.8	**	11.9	**	4.3	*	1.5	***	31.8	***	312.4	***	9.7	**	105.1	***	2.0	ns	85.3	***
S17×J	0.1	ns	315.1	***	6.7	**	9.1	***	23.5	***	17.7	***	26.7	***	85.1	***	1.1	ns	8.8	**
Cu×J	238.8	***	100.1	***	53.1	***	19.2	***	115.2	***	332.8	***	63.9	***	137.9	***	61.3	***	39.1	***
S17×Cu×J	138.0	***	36.4	***	30.2	***	142.4	***	16.4	***	232.2	***	7.1	**	203.9	***	1.6	ns	68.7	***
